# Diets containing the highest levels of dairy products are associated with greater eutrophication potential but higher nutrient intakes and lower financial cost in the United Kingdom

**DOI:** 10.1007/s00394-019-01949-y

**Published:** 2019-03-29

**Authors:** D. A. Hobbs, C. Durrant, J. Elliott, D. I. Givens, J. A. Lovegrove

**Affiliations:** 1 Centre of Excellence for Sustainable Food Systems, University of Liverpool, Liverpool Science Park IC2, 146 Brownlow Hill, Liverpool, L3 5RF UK; 2 Department of Food and Nutritional Sciences, Hugh Sinclair Unit of Human Nutrition, University of Reading, Reading, UK; 3 Institute for Cardiovascular and Metabolic Research, University of Reading, Reading, UK; 4 Institute for Food, Nutrition and Health, University of Reading, Reading, UK; 5 ADAS, Wolverhampton, UK

**Keywords:** Dairy, Environmental impact, Diet quality, Cardio-metabolic health

## Abstract

**Purpose:**

Previously, the nutritional contribution, environmental and financial costs of dairy products have been examined independently. Our aim was to determine the nutritional adequacy, financial cost and environmental impact of UK diets according to dairy content.

**Methods:**

In this cross-sectional study of adults (19–64 years) from the UK National Diet and Nutrition Survey years 1–4 (*n* = 1655), dietary intakes assessed from 4-day estimated food diaries were organized into quartiles (Q) total grams of dairy (milk, cheese, yogurt, dairy desserts) and analyzed using ANCOVA controlling for age, sex and energy intake with Bonferroni post hoc test for nutritional adequacy, Alternative Healthy Eating Index (AHEI-2010), environmental impact [greenhouse gas emissions (GHGE), eutrophication and acidification potentials], financial cost, markers of health and cardio-metabolic diseases.

**Results:**

Nutritional adequacy, particularly for protein, calcium and iodine (+ 18 g, + 533 mg, + 95 g, respectively, all *P* < 0.0001) and AHEI-2010 (*P* < 0.0001) were significantly higher and systolic BP (− 2 mmHg, *P* = 0.019) was significantly lower for the higher-dairy diets (Q4, 274–1429 g/day dairy), compared with diets containing lower dairy (Q1, 0–96 g/day dairy). Diets in Q4 had lower financial cost (− 19%, *P* < 0.0001) and the greatest eutrophication potential, compared with Q1 (+ 29%, *P* < 0.0001). Yet the environmental (GHGE) and financial costs per unit nutrient (riboflavin, zinc, iodine, magnesium, calcium, potassium) were lower in Q4 than Q1 (all *P* < 0.0001).

**Conclusion:**

Diets with the highest dairy content had higher nutrient composition, better diet quality, were associated with lower BP and financial cost, but with higher eutrophication potential. Robust environmental data for many of food groups are limited and this needs an urgent addressing.

**Trial registration:**

This trial was registered on clinicaltrials.gov as NCT03407248.

**Electronic supplementary material:**

The online version of this article (10.1007/s00394-019-01949-y) contains supplementary material, which is available to authorized users.

## Introduction

Global population growth, which is estimated to rise to > 9 billion by 2050, is placing greater demand on the planet’s finite natural resources. Estimates suggest that world food demand will increase at an average rate of 1.1% annually between now and 2050 [1]. Supplying the growing population with sufficient food to meet energy and nutrient needs is, therefore, one of the world’s greatest challenges.

The manufacture of food impacts on the environment through, for example, the production of greenhouse gas emissions (GHGE) such as carbon dioxide, methane and nitrous oxide, and the use of land as well as water resources. The environmental impact arises at all stages in the life cycle from the processes of agricultural production, processing, transport, storage, cooking, through to disposal of waste [2]. Estimates suggest that the food system contributes 19–29% of global GHGEs [3] and accounts for 70% of global freshwater use [4]. Animal products, particularly meat and dairy are generally associated with relatively large environmental impacts on a per kg basis. Dietary change along with efficiencies in food production and reductions in food waste is, therefore, an important strategy to reduce environmental impacts of the food system [5].

In the UK, consumption of milk and dairy products has changed substantially over the previous decades. Since the 1970s, milk consumption by adults has fallen from around 2.5 L per person per week to 1.5 L per person per week [6]. Moreover, from around 1990, the quantity of fat-reduced milk consumed has exceeded that of full-fat milk, and the trend of replacing full-fat milk with fat-reduced milk has continued [6]. Over the same period, yogurt and fromage frais consumption has increased substantially, whilst cheese consumption has seen an overall steady increase [6]. Despite these very large changes, milk and dairy products remain an important dietary source of key nutrients for a large proportion of the UK population. For example, in adults, dairy products provide around 50% of the reference nutrient intake (RNI) for calcium and phosphorus, and 40% and 107%, of the RNI for riboflavin and vitamin B_12_, respectively [7]. In addition, for many of the nutrients, dairy products have a high nutrient density meaning that less energy needs to be consumed to provide the same nutrient intake [8].

Milk and dairy products also contribute around 27% of saturated fat intake in the UK diet [7]. Higher dietary saturated fat consumption is associated with an increased risk of cardiovascular disease (CVD), which is largely due to the low-density lipoprotein cholesterol (LDL-C) raising effects of saturated fat [9]. However, evidence from a number of meta-analyses of prospective cohort studies show that the consumption of milk and other dairy products is either associated with a neutral or reduced risk of CVD [10–12], stroke [13], type 2 diabetes [14] and hypertension [15].

Therefore, when assessing the role of dairy products in sustainable diets, it is important to consider not only the environmental impact, but also the nutritional contribution dairy products make to the diet, together with other health beneficial functionality.

The aim of this study was to determine the associations between UK diets containing varying levels of dairy products with nutritional adequacy, dietary cost and GHGE, acidification and eutrophication potentials.

## Methods

### Dietary data

Data files from years 1 to 4 (2008/2009–2011/2012) of the NDNS were obtained from the UK Data Archive (https://www.data-archive.ac.uk) [7]. Overall, the response rate of participants completing three or four diet diary days was 56% in years 1–4 giving a total sample size of 4156 survey participants. In the current analysis, data from the adult population were investigated using the food consumption and nutritional data for all 1655 adults aged 19–64 years (males: *n = *710 and females: *n = *945), unless otherwise stated. The mean quantity of individual foods consumed per day was aggregated into food sub-groups (136 food sub-groups, excluding dietary supplements, commercial toddler food and drink, artificial sweeteners and nutrition powders and drinks) according to the NDNS classification. Non-consumers of each food group were included in the analysis. The dairy food group included milk, cheese, yogurt, fromage frais, ice cream, other dairy, cream and dairy desserts, but not butter as this is classified as part of the fats and oils food group in NDNS. Milk alternatives such as almond, rice and soya milks were removed from the food groups and analyzed as a separate food group. The nutritional contribution of each food group to average estimated requirement (EAR) for energy, dietary reference values (DRV) for macronutrients and reference nutrient intakes (RNI) for vitamins and minerals was also calculated. All micronutrients reported in the NDNS were used in the analysis. The NDNS was conducted according to the guidelines laid down in the Declaration of Helsinki, and ethical approval for all procedures was granted by Local Research Ethics Committees covering all areas in the survey. All participants (or where relevant, legal guardians) gave informed consent.

### Assessment of underreporting

Energy misreporting was assessed using Goldberg’s cut-off 2 criterion [16, 17], which uses 95% confidence limits to statistically compare the ratio of reported energy intake (EI) to basal metabolic rate (BMR) with physical activity level (PAL). BMR was estimated using the Henry equation [18] and a PAL of 1.2, representing that a sedentary lifestyle was chosen for the total population. The within-subject variation in reported energy intake (CV_wEI_) and repeated BMR measurements (CV_wBMR_) was 23% and 8.2%, respectively [17]. The between-subject variation in PAL (CV_tP_) was 15% [16, 17]. Subjects were identified as under-reporters if their reported EI was less than the calculated lower cut-off.

### Environmental data

Estimates of GHGEs, eutrophication potential and acidification potential were used to calculate the environmental impact of diets containing varying levels of dairy products. Briefly, data for GHGEs, eutrophication potential and acidification potential associated with each of the 136 sub-food groups from the NDNS were collected during the period of June–September 2014, from relevant literature from the UK and other European countries and were cross-referenced with at least one other source to ensure representativeness. Data were collected from primary production to retail using life-cycle assessment (LCA). Within each NDNS sub-food group, environmental data on the most commonly consumed foods were averaged to produce a single value for each sub-food group. For some composite dishes or processed foods, where a single value was not available, the environmental impact was estimated using component ingredients. This was particularly the case for eutrophication and acidification potentials where data were only available for main food items [19–26]. A detailed discussion of assumptions made can be found in the Supplemental Environmental Methods. A list of GHGE values and data sources used can be found in Supplemental Table 1.

### Cost of diets

The monetary cost of the diets was estimated by collecting UK retail prices of all food items (*n* = 3420) reported in years 1–4 of the NDNS. Briefly, the retail price of food items was collected online from Asda and Waitrose supermarkets during the period of June–September 2014, and was updated in July 2015. The collection of food item costs was standardized, and whenever possible, the minimum weight of a food or product as sold was used. Furthermore, any offers or multi-buys were avoided to ensure that the true cost of a food item was captured. The majority of costs was based on own brand products/house brands for Asda and Waitrose supermarkets. The retail price of each of the food items was calculated as an average cost between Asda and Waitrose, and was aggregated into food groups as described above. In the case of food groups with a large number of individual foods, we took the average cost of the most consumed foods by weight within that food group. A table showing a summary of the food prices used for each sub-food group can be found in Supplemental Table 2.

### Calculation of GHGEs and financial cost (£) of diets per unit nutrient

The GHGEs and financial cost (£) of diets per unit of nutrient were calculated using the following equations:

$$ {\text{GHGE}}\,{\text{per}}\,{\text{unit}}\,{\text{nutrient}} = \frac{{{\text{gCO2e}}\,{\text{per}}\,{\text{day}}}}{{{\text{ug,}}\,{\text{mg}}\,{\text{or}}\,{\text{g}}\,{\text{nutrient}}\,{\text{per}}\,{\text{day}}}} $$

$${\text{Diet}}\,{\text{cost}}\,(\pounds)\,{\text{per}}\,{\text{unit}}\,{\text{nutrient}} = \frac{{{\text{diet}}\,{\text{cost}}\,{\text{per}}\,{\text{day}}}}{{{\text{ug,}}\,{\text{mg}}\,{\text{or}}\,{\text{g}}\,{\text{nutrient}}\,{\text{per}}\,{\text{day}}}}.$$

A cut-off was also applied when 100% of the RNI was met for a particular nutrient. For example, if the unit (µg, mg, g) consumed per day of a nutrient exceeded the RNI for that particular nutrient, then the RNI was used in the calculation instead of the unit nutrient per day.

### Biomarkers of health data

Anthropometric (weight, height, body mass index, waist and hip circumference), blood pressure and blood biomarker data (total cholesterol, high-density lipoprotein cholesterol (HDL-C), low-density lipoprotein cholesterol (LDL-C), triacylglycerol (TAG), C-reactive protein, glycated hemoglobin and glucose) were obtained for participants in the NDNS. Details of how blood samples were taken, stored and analyzed have been described in detail elsewhere [7].

### Food group analysis and calculation of Alternative Healthy Index (AHEI) score

A ‘baseline’ diet was created which was based on the average UK adult (19–64 years) male and female diets from the NDNS. The lower and higher dairy diets were created by splitting the male and female ‘baseline’ diet into quartiles of total grams of dairy, with the lowest quartile representing the lower dairy diet (Q1) and the highest quartile representing the higher dairy diet (Q4). The Alternative Healthy Eating Index (AHEI) score was calculated for each of the diets using the methods previously described [27] with minor modification (Supplemental Table 3). The AHEI score was used in this analysis because previous studies have shown that it is a better predictor of risk of chronic disease compared with other diet scores such as the Healthy Eating Index (HEI)-2010 [27].

### Statistical analysis

The primary outcome of this study was to determine the associations between UK diets containing varying levels of dairy products with nutritional adequacy, dietary cost and three elements of environmental impact (GHGE, acidification and eutrophication potentials). The secondary outcome was to calculate GHGE and financial cost per unit nutrient to estimate the quality costs of each diet. Analysis of covariance (ANCOVA) was used to detect statistically significant differences between diets based on dairy quartiles and nutrient intakes, environmental impact, dietary cost, food groups and GHGEs and financial cost per nutrient controlling for age, sex and total energy intake (kJ). We controlled for total energy intake in our analysis of the environmental impact because GHGEs have been shown to be positively associated with total energy intake [28]. Bonferroni post hoc tests were used to detect differences between quartiles. Significant differences between categorical variables across dairy quartiles were analyzed using Chi-square test for independence. The strength of the association between categorical variables (effect size) was calculated using Cramer’s V (*V*) test or partial Eta^2^ (*η*^2^) for non-categorical variables. We regarded the *P* value of  0.05 as statistically significant. All statistical analyses were performed in SPSS version 21 (SPSS Inc.). In this population, it was estimated that 13% (*n* = 194) of subjects were classed as under-reporters of energy intake. However, underreporting was not considered further in statistical analysis. LRNI was used in this study as it represents a diet that will be insufficient for 97.5% of the population and is also used in NDNS as a marker of diet quality.

## Results

### Sociodemographic and health characteristics of adults by dairy quartile

The sociodemographic and health characteristics of adults by dairy quartile are presented in Table 1. There were significant differences across quartiles for age (*P* = 0.005, partial *η*^2^ = 0.030), qualifications (*P* = 0.010, *V* = 0.0.072), socio-economic classification (*P* = 0.017, *V* = 0.068) ethnic group (*P* = 0.0001, *V* = 0.095) and smoking status (*P* = 0.0001, *V* = 0.113).

**Table 1:** Sociodemographic and health characteristics of British adults, by quartile of dairy intake

	Total *n* = 1655	Quartiles of dairy product consumption (g/day)				*P*
		1 (0–96) *n* = 411	2 (97–172) *n* = 410	3 (173–273) *n* = 414	4 (274–1429) *n* = 420	
Age, (year)	42.7 ± 12.5	39.2 ± 12.5	42.4 ± 12.3	44.5 ± 12.3	44.7 ± 11.9	0.005
Males, (%)	43	42	41	41	48	NS
Qualifications, (%)						0.010
No qualifications (or in full-time education)	19	20	20	19	16	
School certificates and other qualifications	42	49	39	38	44	
Higher education below degree level	27	21	29	29	28	
Degree	12	10	11	14	12	
Equalized annual household income, (£)	33,601 ± 24,737	31,078 ± 25,160	34,581 ± 25,521	32,823 ± 24,409	35,819 ± 23,677	NS
Socio-economic classification, (%)^a^						0.017
Managerial and professional	43	37	44	43	47	
Intermediate and small businesses	28	28	27	31	26	
Routine and never worked	29	35	29	25	27	
Ethnic group, (%)						0.0001
White	90	83	90	91	96	
Black or black British	3	7	3	2	1	
Asian or Asian British	4	5	4	4	2	
Any other group incl mixed	3	5	3	2	2	
Smoking status, (%)						0.0001
Non-smoker	55	14	21	22	23	
Ex-smoker	20	50	54	59	56	
Current smoker	25	36	24	19	21	
Has longstanding illness, yes	33	7	8	9	9	NS

Values are means ± SD or percentages unless otherwise stated. Values shown for quartiles of total dairy product consumption are min and max grams consumed per day. Differences between dairy quartiles for continuous variables were assessed using ANOVA and for categorical variables Chi-square test for independence was used

*NS* not significant

^a^Based on National Statistics Socio-Economic Classification [49]

### Food group analysis and AHEI-2010 score

Diets for the total population and for different quartiles of dairy intake are shown in Table 2. There was a significant difference in consumption of high-fiber breakfast cereals, other breakfast cereals, milk, other milk and cream, cheese, ice cream, yogurt and fromage frais, fruit and tea, coffee and water, alcoholic beverages, soft drinks (not low calorie), chips, milk alternatives (all *P* < 0.0001), bread (*P* = 0.001), vegetables and potatoes (*P* = 0.014), biscuits (*P* = 0.02), eggs and dishes (*P* = 0.039) and preserves and sweet spreads (*P* = 0.004) across quartiles of dairy intake. There were no significant differences in red meat (*P* = 0.18) or processed meat (*P* = 0.20) across increasing quartiles of dairy intake. Bonferroni post hoc analysis comparing the highest (Q4) with the lowest (Q1) dairy quartiles showed that subjects in Q4 had significantly higher intakes of high-fiber breakfast cereals, other breakfast cereals, milk, other milk and cream, cheese, ice cream, yogurt and fromage frais, fruit, tea, coffee and water (all *P* < 0.0001), bread (*P* = 0.001), vegetables and potatoes (*P* = 0.007) and preserves and sweet spreads (*P* = 0.002) and significantly lower intakes of milk alternatives (*P* < 0.0001), eggs and egg dishes (*P* = 0.031), chips (*P* = 0.001), soft drinks (not low calorie) (*P* < 0.0001) and alcoholic beverages (*P* < 0.0001) compared to subjects in Q1.

**Table 2:** Food groups and total AHEI-2010 score according to quartiles of total dairy intake

Food groups, (g/day)	Total *n* = 1655	Quartiles of total dairy product consumption (g/day)				*P*^a^ Q1 vs. Q4	*P*^b^
		1 (0–96) *n* = 411	2 (97–172) *n* = 410ssss	3 (173–273) *n* = 414	4 (274–1429) *n* = 420		
Total AHEI score (out of 110)	56 (55, 56)	53 (52, 54)	56 (55, 57)	57 (55, 58)	58 (57, 59)	< 0.0001	< 0.0001
Cereals and cereal products							
Pasta, rice and other cereals	78 (74, 82)	83 (74, 92)	75 (68, 83)	79 (71, 87)	76 (68, 84)	0.14	0.14
Bread	84 (81, 86)	84 (79, 89)	83 (78, 88)	81 (77, 86)	87 (82, 91)	0.001	0.001
High-fiber breakfast cereals	20 (18, 22)	7.7 (5.4, 10)	15 (12, 18)	22 (19, 26)	34 (27, 40)	< 0.0001	< 0.0001
Other breakfast cereals	5.5 (5.0, 6.1)	2.1 (1.5, 2.6)	4.7 (3.8, 5.6)	6.9 (5.7, 8.1)	8.5 (7.0, 10)	< 0.0001	< 0.0001
Puddings	11.2 (9.9, 12.5)	7.8 (5.8, 9.8)	9.7 (7.0, 12)	11 (8.8, 14)	16 (13, 19)	0.34	0.26
Biscuits (cookies)	14 (13, 15)	10 (8.7, 12)	13 (11, 14)	16 (14, 18)	16 (14, 18)	0.20	0.02
Buns, cakes, pastries	18 (16, 19)	13 (10, 15)	16 (13, 18)	19 (17, 22)	23 (20, 26)	1.0	0.51
Milk and milk products							
Milks	140 (134, 146)	35 (32, 38)	88 (85, 92)	146 (140, 151)	288 (273, 303)	< 0.0001	< 0.0001
Other milk and cream	8.4 (6.9, 9.9)	2.5 (1.8, 3.3)	4.6 (3.1, 6.2)	9.7 (6.8, 13)	17 (12, 21)	< 0.0001	< 0.0001
Cheese	15 (14, 16)	8.6 (7.3, 9.8)	13 (12, 15)	17 (15, 19)	20 (18, 22)	< 0.0001	< 0.0001
Ice cream	5.1 (4.5, 5.8)	2.9 (2, 3.8)	3.8 (2.7,4.9)	5.6 (4.4, 6.8)	8.1 (6.4, 9.8)	< 0.0001	< 0.0001
Yogurt, fromage frais	29 (26, 31)	5.0 (3.5, 6.5)	19 (16, 22)	31 (26, 35)	59.6 (52.8, 66.5)	< 0.0001	< 0.0001
Milk alternatives	2.5 (1.5, 3.5)	5.8 (2.7, 8.8)	2.8 (0.8, 4.9)	0.9 (0.7, 2.4)	0.7 (0.1, 1.5)	< 0.0001	< 0.0001
Eggs and dishes	20 (18, 22)	22 (17, 27)	18 (15, 21)	21 (18, 24)	20 (17, 23)	0.031	0.039
Fat spreads and oils	11 (10, 11)	11 (9.5, 11)	9.8 (9.0, 11)	11 (9.8, 11)	12 (11, 13)	0.71	0.21
Meat and meat products							
Red meat	71 (67, 75)	69 (61, 77)	67 (60, 75)	77 (68, 86)	70 (62, 79)	0.59	0.18
Processed meat	45 (43, 48)	45 (41, 50)	44 (39, 49)	41 (36, 46)	50 (44, 55)	1.00	0.20
Chicken and turkey	64 (60, 67)	67 (58, 75)	66 (59, 73)	61 (54, 67)	62 (55, 68)	0.34	0.15
Other meat and offal	6.6 (5.5, 7.8)	6.6 (4.5, 8.8)	6.6 (4.3, 8.9)	5.5 (3.5, 7.6)	7.7 (5.2, 10)	1.00	0.44
Fish and dishes							
Oily fish	11 (9.6, 12)	10 (7.1, 14)	12 (8.7, 14)	11 (8.7, 13)	11 (8.6, 14)	1.0	0.46
White fish coated or fried	8.3 (7.4, 9.2)	9.5 (7.7, 11)	7.7 (5.9, 9.4)	7.7 (6.0, 9.3)	8.4 (6.7, 10)	0.36	0.16
Other white fish, shellfish	18 (16, 20)	16 (12, 20)	18 (14, 21)	20 (16, 25)	19 (15, 23)	1.0	0.76
Vegetables and potatoes	180 (175, 186)	156 (145, 166)	177 (165, 189)	183 (172, 194)	205 (193, 217)	0.007	0.014
Chips	40 (38, 42)	46 (41, 50)	38 (34, 42)	37 (33, 41)	39 (35, 44)	0.001	< 0.0001
Savory snacks	7.2 (6.7, 7.7)	7.5 (6.4, 8.6)	7.2 (6.1, 8.2)	7.4 (6.4, 8.5)	6.8 (5.8, 7.8)	0.26	0.11
Nuts and seeds	2.6 (2.2, 3.0)	1.8 (1.2, 2.5)	2.8 (2.0, 3.7)	2.6 (1.8, 3.5)	3.2 (2.3, 4.0)	1.0	0.63
Fruit	94 (89, 99)	67 (59, 76)	90 (81, 100)	98 (89, 108)	119 (108, 131)	< 0.0001	< 0.0001
Sugar, preserves, confectionery							
Chocolate confectionery	8.4 (7.7, 9.2)	7.2 (5.9, 8.5)	8.7 (7.1, 10.3)	8.6 (7.1, 10.2)	9.3 (7.6, 11)	1.0	0.25
Sugar confectionery	1.8 (1.4, 2.1)	1.5 (0.9, 2.1)	1.7 (1.0, 2.5)	1.8 (1.0, 2.5)	2.0 (1.2, 2.7)	1.0	0.98
Sugar, preserves, sweet spreads	11 (10, 12)	8.4 (7.2, 9.6)	11 (9.2, 12)	12 (10, 13)	14 (12, 16)	0.002	0.004
Non-alcoholic beverages							
Fruit juice	53 (47, 58)	51 (39, 62)	52 (43, 62)	50 (39, 61)	57 (45, 69)	1.0	0.76
Soft drinks (low calorie)	108 (97, 120)	131 (104, 159)	109 (87, 130)	94 (75, 112)	100 (77, 123)	1.0	0.41
Soft drinks (not low calorie)	123 (112,133)	161 (134, 188)	115 (94, 137)	122 (101, 142)	93 (78, 108)	< 0.0001	< 0.0001
Tea, coffee and water	1113 (1083, 1143)	940 (876, 1004)	1030 (974, 1085)	1200 (1146, 1254)	1279 (1217, 1341)	< 0.0001	< 0.0001
Alcoholic beverages	267 (243, 292)	307 (258, 357)	298 (244, 353)	231 (187, 276)	234 (185, 283)	< 0.0001	< 0.0001
Miscellaneous foods	59 (55, 62)	62 (54, 70)	60 (53, 67)	54 (47, 61)	59 (52, 66)	0.45	0.12

Values are non-adjusted means (95% CIs). Values shown for quartiles of total dairy product consumption are min and max grams consumed per day. Total dairy products AHEI, Alternative Healthy Eating Index

^a^Based on Bonferroni post hoc test comparing the highest (Q4) and lowest (Q1) dairy quartiles

^b^Differences between food groups across dairy quartiles using general linear models adjusted for age, sex and total energy intake

There was a significant difference in AHEI across dairy quartiles (*P* < 0.0001), with the diets of adults in Q2 (*P* = 0.022), Q3 (*P* < 0.0001) and Q4 (*P* < 0.0001) having significantly higher AHEI scores compared with the diets of adults in Q1 (Table 2).

### Nutrient intakes and adequacy of diets

The nutrient intakes and adequacy of the total population and across increasing quartiles of dairy intake are shown in Table 3. When controlling for age, sex and total energy intake (kJ), there was a significant increase in total energy intake, carbohydrate, protein, saturated fat, *cis*-MUFA, PUFA, calcium, magnesium, potassium, iodine, zinc, thiamine, riboflavin, vitamin B_12_, folate (all *P* < 0.0001) and iron (*P* = 0.014) across increasing quartiles of dairy intake. For total energy intake, carbohydrate, protein, saturated fat, *cis*-MUFA, PUFA, calcium, magnesium, potassium, iodine, zinc, thiamine, riboflavin, vitamin B_12_, folate and iron, there was a significantly higher intake by adults in Q4 compared with Q1 (all *P* < 0.0001, except *cis*-MUFA (*P* = 0.001). In addition, for the nutrients that were significantly different across dairy quartiles (vitamin B_12_, riboflavin, calcium, iodine, folate, zinc, magnesium, iron and potassium), the percentage of subjects below the LRNI was less in Q4 compared with Q1 (Table 3). For thiamine, no participants were below the LRNI across dairy quartiles.

**Table 3:** Nutrient intakes and macronutrient contributions to EARs and DRVs and (%) of participants below LRNIs for micronutrients according to quartiles of total dairy intake

Intakes per day^a^		Quartiles of total dairy product consumption (g/day)				*P*^b^ Q1 vs. Q4	*P*^c^
	Total *n* = 1655	1 (0–96) *n* = 411	2 (97–172) *n* = 410	3 (173–273) *n* = 414	4 (274–1429) *n* = 420		
Energy, (MJ)	7.7 (7.6, 7.8)	7.1 (6.8, 7.3)	7.3 (7.1, 7.5)	7.7 (7.5, 7.9)	8.7 (8.4, 8.9)	< 0.0001	< 0.0001
(%) of EAR	78	71	75	79	88		
Carbohydrate, (g)	219 (216,223)	197 (190, 204)	207 (199, 212)	224 (217, 230)	251 (244, 258)	< 0.0001	< 0.0001
(%) of DRV	91	90	90	92	92		
NMES, (g)	57 (55, 59)	58 (49, 56)	53 (50, 57)	58 (54,62)	64 (60,68)	1.0	0.48
(%) of DRV	93	105	110	109	107		
Fat, (g)	68 (66, 69)	62 (59, 64)	63 (61, 66)	68 (66, 71)	77 (75, 80)	1.0	0.74
(%) of DRV	99	100	99	99	99		
Saturated fat, (g)	25 (24, 25)	21 (20, 22)	23 (22, 24)	27 (25, 27)	30 (29, 31)	< 0.0001	< 0.0001
(%) of DRV	116	109	113	118	123		
*cis*-MUFA, (g)	25 (24,25)	23 (22,24)	23 (22,24)	25 (24, 26)	27 (26,28)	0.001	0.003
(%) of DRV	97	101	97	96	94		
PUFA, (g)	12 (12, 12)	12 (11, 12)	11 (11, 12)	12 (11, 12)	13 (12, 13)	< 0.0001	< 0.0001
(%) of DRV	93	100	96	90	87		
Protein, (g)	74 (72, 75)	67 (64, 70)	69 (67, 72)	73 (71, 75)	85 (83, 88)	< 0.0001	< 0.0001
(%) of RNI	148	134	142	150	172		
Calcium, (mg)	806 (791, 821)	580 (558, 601)	694 (674, 713)	830 (809, 850)	1113 (1085, 1141)	< 0.0001	< 0.0001
(%) below LRNI	7	21	4	1	0		
Magnesium, (mg)	253 (249, 258)	221 (212, 230)	237 (229, 244)	255 (248, 263)	300 (291, 308)	< 0.0001	< 0.0001
(%) below LRNI	13	25	15	8	4		
Sodium, (mg)	2254 (2213, 2295)	2134 (2050, 2217)	2148 (2072, 2224)	2247 (2167, 2327)	2483 (2400, 2566)	1.0	0.81
(%) below LRNI	1	2	0	0	0		
Potassium, (mg)	2787 (2744, 2830)	2386 (2304, 2469)	2589 (2513, 2665)	2816 (2745, 2888)	3344 (3261, 3427)	< 0.0001	< 0.0001
(%) below LRNI	17	31	23	11	3		
Iron, (mg)	11 (10, 11)	9.5 (9.1, 9.9)	10 (9.8, 10)	11 (11, 11)	12 (12, 12)	0.063	0.014
(%) below LRNI	14	25	17	9	6		
Selenium, (µg)	48 (47, 49)	46 (44, 49)	46 (44, 48)	47 (45, 48)	51 (49, 53)	0.14	0.08
(%) below LRNI	40	46	44	40	30		
Iodine, (µg)	161 (158, 165)	122 (115, 129)	141 (135, 147)	164 (158, 170)	217 (209, 224)	< 0.0001	< 0.0001
(%) below LRNI	7	22	6	1	0		
Zinc, (mg)	8.5 (8.4, 8.7)	7.6 (7.2, 7.9)	7.9 (7.7, 8.2)	8.6 (8.3, 8.8)	10 (9.8, 10)	< 0.0001	< 0.0001
(%) below LRNI	6	14	7	4	1		
Thiamine, (mg)	1.4 (1.4, 1.4)	1.2 (1.2, 1.3)	1.3 (1.3, 1.4)	1.4 (1.4, 1.5)	1.7 (1.6, 1.7)	< 0.0001	< 0.0001
(%) below LRNI	0	0	0	0	0		
Riboflavin, (mg)	1.6 (1.5, 1.6)	1.1 (1, 1.2)	1.3 (1.3, 1.4)	1.6 (1.6, 1.7)	2.1 (2.1, 2.2)	< 0.0001	< 0.0001
(%) below LRNI	8	26	4	0	0		
Niacin (eqv, mg)	36 (35, 37)	34 (32, 36)	35 (34, 36)	36 (35, 37)	40 (38, 41)	1.0	0.71
(%) below LRNI	0	0	0	0	0		
Vitamin B_6_, (mg)	2.2 (2.1, 2.2)	2 (1.9, 2.1)	2.1 (2, 2.2)	2.1 (2.1, 2.2)	2.5 (2.4, 2.6)	0.28	0.12
(%) below LRNI	7	13	9	5	2		
Vitamin B_12_, (µg)	5.2 (5, 5.4)	4.1 (3.8, 4.4)	4.6 (4.2, 5)	5.3 (4.9, 5.6)	6.7 (6.4, 7)	< 0.0001	< 0.0001
(%) below LRNI	1	5	0	0	0		
Folate, (µg)	256 (251, 261)	221 (211, 231)	242 (233, 251)	262 (252, 271)	297 (286, 309)	< 0.0001	< 0.0001
(%) below LRNI	3	8	3	2	1		
Vitamin C, (mg)	83 (80, 87)	71 (65, 78)	85 (79, 91)	84 (78, 90)	93 (87, 100)	0.61	0.11
(%) below LRNI	1	3	1	1	0		
Vitamin A^d^, (mg)	961 (916, 1005)	827 (746, 908)	912 (817, 1008)	974 (889, 1059)	1125 (1032, 1218)	0.24	0.21
(%) below LRNI	ss8	15	9	5	2		

Values are non-adjusted means (95% CIs). Values shown for quartiles of total dairy product consumption are min and max grams consumed per day

*EAR* estimated average requirement, *DRV* daily recommended value, *LRNI* lower reference nutrient intake, *RNI* reference nutrient intake, *NMES* non-milk extrinsic sugars

^a^Percentage contribution to ADIs for: carbohydrate (50% total dietary energy); non-milk extrinsic sugars (11% food energy); fat (35% food energy); saturated fat (11% food energy); polyunsaturated fat (6.5% food energy) and monounsaturated fat (13% food energy)

^b^Based on Bonferroni post hoc test comparing the highest (Q4) and lowest (Q1) dairy quartiles

^c^Differences between nutrient intakes across dairy quartiles using general linear models adjusted for age, sex and total energy intake

^d^Retinol equivalents

### Environmental impact of diets

When controlling for age, sex and total energy intake (kJ), there was a significant difference in eutrophication potential across increasing quartile of dairy intake (non-adjusted and adjusted values *P* < 0.0001) with the diets containing the highest amount of dairy (Q4) having significantly higher eutrophication potential (29%) (all *P* < 0.0001) compared with the diets containing the lowest amount of dairy (Q1, Table 4).

**Table 4:** Environmental impacts and diet cost for the total population and for quartiles of total dairy intake

Environment and diet cost per day		Quartiles of total dairy product consumption (g/day)				*P*^a^ Q1 vs. Q4	*P*^b^
	Total *n* = 1655	1 (0–96) *n* = 411	2 (97–172) *n* = 410	3 (173–273) *n* = 414	4 (274–1429) *n* = 420		
GHGE, (kg CO_2_ eqv)							
Non-adjusted values	4.1 (4.0–4.1)	3.7 (3.5, 3.8)	3.9 (3.8, 4.0)	4.1 (3.9, 4.2)	4.6 (4.5, 4.8)	< 0.0001	< 0.0001
Adjusted values		4.0 (3.9, 4.1)	4.1 (4.0, 4.2)	4.0 (3.9, 4.1)	4.1 (4.0, 4.2)	1.00	1.00
Eutrophication, (g N eqv)							
Non-adjusted values	54.0 (52.3–55.7)	46.9 (43.7, 50.0)	50.3 (47.3, 53.4)	53.3 (50.1, 56.4)	65.3 (61.7, 68.9)	< 0.0001	< 0.0001
Adjusted values		50.8 (47.7, 54.0)	51.9 (48.7, 55.0)	52.5 (49.4, 55.6)	60.7 (57.5, 63.9)	< 0.0001	< 0.0001
Acidification, (g SO_2_ eqv)							
Non-adjusted values	35.2 (34.4–36.0)	33.3 (31.2, 35.4)	32.9 (31.4, 34.3)	34.4 (33.0, 35.7)	40.0 (38.5, 41.5)	< 0.0001	< 0.0001
Adjusted values		35.9 (34.5–37.3)	34.4 (33.1–35.8)	34.2 (32.9–35.6)	36.1 (34.8–37.5)	1.00	0.081
Dietary cost, (£)							
Non-adjusted values	5.3 (5.2–5.4)	5.2 (4.9, 5.4)	5.3 (5.0, 5.5)	5.1 (4.9, 5.4)	5.6 (5.3, 5.8)	0.14	0.045
Adjusted values		5.8 (5.6–5.9)	5.6 (5.4–5.8)	5.1 (4.9–5.2)	4.7 (4.6–4.9)	< 0.0001	< 0.0001

All values are mean (95% CIs). Values shown for quartiles of total dairy product consumption are min and max grams consumed per day

*GHGE* greenhouse gas emissions

^a^Based on Bonferroni post hoc test comparing the highest (Q4) and lowest (Q1) dairy quartiles

^b^Differences between environmental impact measure and diet cost across dairy quartiles using general linear models adjusted for age, sex and total energy intake

For GHGE and acidification potential, there was a significant difference across dairy quartiles in the non-adjusted model only (both *P* < 0.0001) with the diets containing the highest amount of dairy (Q4) having significantly higher GHGE and acidification potential (both *P* < 0.0001) compared with the diets containing the lowest amount of dairy (Q1). However, the significance was lost when the analysis was adjusted for energy intake, age and gender (GHGE; *P-*trend = 1.00 and acidification potential; *P*-trend = 0.045, Table 4).

### Cost of diets

There was a significant difference between dietary costs (£/day) across increasing quartile of dairy intake in the non-adjusted model (*P* = 0.045); however, there was no significant difference in dietary cost between Q4 and Q1 (*P* = 0.14, Table 4). In the adjusted model (controlling for age, sex and energy intake), dietary cost was significantly different across dairy quartiles (*P* < 0.0001), with diets in Q4 being on average 19% (mean SEM: £1.1/day 0.09) cheaper than Q1.

### Associations between dairy quartiles and biomarkers of health

There was a significant difference across increasing quartiles of total dairy intake for height (*P* = 0.039) in the non-adjusted model (Table 5). When adjusting for age, sex, BMI and energy intake (model 2), there was a significant difference in SBP (*P* = 0.019) and DBP (*P* = 0.037) across quartiles of total dairy intake, with individuals in Q4 having significantly lower SBP (*P* = 0.028) compared with individuals in Q1. There were no other significant differences between quartiles of total dairy intake and biomarkers of health.

**Table 5:** Differences in biomarkers of health between quartiles of total dairy intake

Biomarkers	Quartiles of dairy product consumption (g/day)				*P *value^a^	
	1 (0–110) *n* = 164	2 (111–188) *n* = 162	3 (189–296) *n* = 165	4 (297–1206) *n* = 170	Model 1	Model 2
Height, (cm)	168 (167, 170)	167 (166, 169)	168 (166, 169)	170 (168, 171)	0.039	0.15
Weight, (kg)	79 (77, 82)	78 (75, 80)	76 (74, 78)	78 (76, 81)	0.26	0.28
BMI, (kg/m^2^)	28 (27, 29)	28 (27, 29)	27 (26, 28)	27 (26, 28)	0.13	0.25
Waist circumference, (cm)	93 (91, 95)	93 (91, 95)	91 (89, 93)	91 (89, 93)	0.27	0.18
Hip circumference, (cm)	107 (106, 109)	106 (105, 108)	105 (104, 107)	105 (104, 107)	0.13	0.15
Waist–hip ratio	0.86 (0.85, 0.88)	0.87 (0.86, 0.89)	0.86 (0.85, 0.87)	0.86 (0.85, 0.88)	0.48	0.27
SBP, (mmHg)	126 (124, 129)	125 (122, 127)	126 (123, 128)	124 (122, 126)	0.38	0.019
DBP, (mmHg)	76 (74, 78)	75 (73, 76)	76 (74, 77)	73 (72, 75)	0.12	0.037
PP, (mmHg)	71 (69, 73)	71 (69, 72)	71 (69, 72)	70 (68, 72)	0.83	0.95
Total-C, (mmol/L)	5.3 (5.1, 5.5)	5.2 (5.1, 5.4)	5.3 (5.1, 5.5)	5.3 (5.1, 5.5)	0.89	0.48
HDL-C, (mmol/L)	1.2 (1.1, 1.4)	1.3 (1.1, 1.4)	1.3 (1.1, 1.4)	1.3 (1.2, 1.4)	0.85	1.00
LDL-C, (mmol/L)	1.5 (1.5, 1.6)	1.5 (1.4, 1.6)	1.5 (1.4, 1.6)	1.6 (1.5, 1.6)	0.30	0.24
Total-C:HDL-C ratio	3.2 (3.1, 3.4)	3.2 (3.1, 3.3)	3.3 (3.1, 3.4)	3.2 (3, 3.3)	0.88	0.56
TAG, (mmol/L)	3.6 (3.4, 3.7)	3.7 (3.5, 3.9)	3.7 (3.5, 3.9)	3.6 (3.4, 3.8)	0.64	0.55
CRP, (mg/L)	3.1 (2.4, 3.8)	2.8 (2.3, 3.3)	3.3 (2.5, 4.1)	2.7 (2.3, 3.1)	0.55	0.62
HbA1c, (%)	5.4 (5.4, 5.5)	5.5 (5.4, 5.6)	5.5 (5.4, 5.6)	5.6 (5.5, 5.7)	0.07	0.21
Glucose, (mmol/L)	5.1 (5, 5.2)	5.1 (5, 5.2)	5.2 (5, 5.3)	5.2 (5, 5.5)	0.63	0.77

Values are non-adjusted means (95% CIs). Values shown for quartiles of total dairy product consumption are min and max grams consumed per day. Please note the sample size (*n* = 661), which is due to a large number of missing samples (*n* = 994)

*CRP* C-reactive protein, *DBP* diastolic blood pressure, *HbA1c* glycated hemoglobin, *HDL-C* high-density lipoprotein cholesterol, *LDL-C* low-density lipoprotein cholesterol, *PP* pulse pressure, *SBP* systolic blood pressure, *TAG* triacylglycerol, *Total-C* total cholesterol

^a^Differences between health biomarkers across dairy quartiles using general linear models. Model 1 was non-adjusted and model 2 was adjusted for age, sex and energy intake with additional adjustment of BMI for systolic, diastolic and pulse pressure

### GHGE and financial cost of diets per nutrient

The GHG emissions per unit nutrient with a cut-off of 100% RNI for each nutrient were significantly different across quartiles of dairy intake for riboflavin (*P* < 0.0001), calcium (*P* < 0.0001), magnesium (*P* = 0.013), potassium (*P* < 0.0001), zinc (*P* = 0.018) and iodine (*P* < 0.0001) (Table 6). Bonferroni post hoc test revealed that the GHGEs per unit nutrient were significantly less for riboflavin (3%, Q4 vs. Q1 *P* = 0.0001), calcium (6%, Q4 vs. Q1 = 0.0001), magnesium (0.3%, Q4 vs. Q1 *P* = 0.025), potassium (6%, Q4 vs. Q1 *P* = 0.0001) and iodine (14%, Q4 vs. Q1 *P* = 0.0001) and significantly higher for zinc (3%, Q4 vs. Q1 *P* = 0.039) in diets in Q4 compared to diets in Q1.

**Table 6:** Average GHGE (g CO_2_ eqv) produced per mg or µg nutrient by quartiles of total dairy intake among British adults

Nutrients	Quartiles of dairy product consumption (g/day)				*P*^a^ Q1 vs. Q4	*P*^b^
	1 (0–96) 411	2 (97–172) 410	3 (173–273) 414	4 (274–1429) 420		
Riboflavin, (mg)	4688 (4454, 4922)	4113 (3966, 4260)	4135 (4005, 4265)	4563 (4433, 4693)	< 0.0001	< 0.0001
Calcium, (mg)	8.3 (8.0, 8.6)	7.4 (7.1, 7.7)	7.2 (7.0, 7.4)	7.8 (7.6, 8.1)	< 0.0001	< 0.0001
Magnesium, (mg)	21 (20, 22)	20 (20, 21)	20 (20, 21)	21 (20, 22)	0.025	0.013
Potassium, (mg)	1.9 (1.8, 2.0)	1.8 (1.8, 1.9)	1.8 (1.7, 1.8)	1.8 (1.7, 1.8)	< 0.0001	< 0.0001
Zinc, (mg)	673 (644, 701)	657 (634, 679)	661 (642, 681)	693 (675, 711)	0.039	0.018
Iodine, (µg)	46 (44, 49)	40 (38, 41)	37 (36, 39)	40 (38, 41)	< 0.0001	< 0.0001

All values are non-adjusted mean (95% CIs). Values shown for quartiles of total dairy product consumption are min and max grams consumed per day. The variable was calculated by dividing GHGE (g CO_2_ eqv) per day by nutrient intakes per day with a cut-off when 100% of the RNI was met for each nutrient. Data shown for nutrients with significant differences (*P* < 0.05) across quartiles of dairy intake. No significant differences were found for energy, protein, fat, SFA, vitamin A, thiamine, niacin, vitamin B_6_, vitamins B_12_, folate, vitamin C, iron, selenium and sodium across quartiles of dairy intake

*GHGE* greenhouse gas emissions

^a^Based on Bonferroni post hoc test comparing the highest (Q4) and lowest (Q1) dairy quartiles

^b^Differences between GHGE per unit nutrient across dairy quartiles using general linear models adjusted for age, sex and total energy intake

The financial cost per unit nutrient with a cut-off of 100% RNI for each nutrient was significantly different across dairy quartiles for energy intake, protein, SFA, thiamine, riboflavin, vitamin B_12_, folate, iron, calcium, magnesium, potassium, zinc and iodine (all *P* < 0.0001; Table 7). Bonferroni post hoc test revealed that the dietary cost per unit nutrient was significantly (Q4 vs. Q1 all *P* = 0.0001) less in Q4 for energy intake (9%), protein (1%), SFA (20%), riboflavin (17%), folate (4%), iron (4%), calcium (19%), magnesium (14%), potassium (19%), zinc (13%) and iodine (25%) and significantly higher for thiamine (2%) and vitamin B_12_ (3%) compared with Q1.

**Table 7:** Average cost per unit of nutrient in GBP (£) across quartiles of total dairy intake among British adults

Participants, (*n*)	Quartiles of dairy product consumption (g/day)				*P*^a^ Q1 vs. Q4	*P*^b^
	1 (0–96) 411	2 (97–172) 410	3 (173–273) 414	4 (274–1429) 420		
Energy, (MJ)	0.76 (0.73, 0.78)	0.74 (0.72, 0.76)	0.69 (0.67, 0.71)	0.69 (0.66, 0.71)	*P* < 0.0001	*P* < 0.0001
Protein, (g)	0.11 (0.11, 0.12)	0.11 (0.10, 0.11)	0.11 (0.10, 0.11)	0.11 (0.11, 0.12)	*P* < 0.0001	*P* < 0.0001
SFA, (g)	0.30 (0.29, 0.32)	0.28 (0.27, 0.30)	0.26 (0.25, 0.27)	0.24 (0.23, 0.25)	*P* < 0.0001	*P* < 0.0001
Thiamine, (mg)	6.3 (6.0, 6.6)	6.2 (6.0, 6.5)	6.0 (5.8, 6.3)	6.4 (6.1, 6.7)	*P* < 0.0001	*P* < 0.0001
Riboflavin, (mg)	5.6 (5.3, 6.0)	4.7 (4.6, 4.9)	4.4 (4.3, 4.6)	4.7 (4.5, 4.9)	*P* < 0.0001	*P* < 0.0001
Vitamin B_12,_ (µg)	3.6 (3.5, 3.8)	3.6 (3.4, 3.8)	3.5 (3.3, 3.6)	3.8 (3.6, 3.9)	*P* < 0.0001	*P* < 0.0001
Folate, (µg)	0.030 (0.029, 0.032)	0.029 (0.028, 0.030)	0.027 (0.026, 0.028)	0.029 (0.028, 0.030)	*P* < 0.0001	*P* < 0.0001
Iron, (mg)	0.66 (0.63, 0.69)	0.63 (0.60, 0.66)	0.59 (0.56, 0.61)	0.64 (0.61, 0.67)	*P* < 0.0001	*P* < 0.0001
Calcium, (mg)	0.010 (0.0095, 0.0104)	0.0085 (0.0082, 0.0089)	0.0077 (0.0074, 0.008)	0.0081 (0.0077, 0.0084)	*P* < 0.0001	*P* < 0.0001
Magnesium, (mg)	0.025 (0.024, 0.026)	0.023 (0.022, 0.024)	0.021 (0.021, 0.022)	0.021 (0.021, 0.022)	*P* < 0.0001	*P* < 0.0001
Potassium, (mg)	0.0022 (0.0022, 0.0023)	0.0021 (0.0020, 0.0021)	0.0019 (0.0018, 0.0019)	0.0018 (0.0017, 0.0019)	*P* < 0.0001	*P* < 0.0001
Zinc, (mg)	0.81 (0.77, 0.86)	0.76 (0.73, 0.80)	0.71 (0.68, 0.74)	0.71 (0.68, 0.73)	*P* < 0.0001	*P* < 0.0001
Iodine, (µg)	0.054 (0.051, 0.058)	0.045 (0.043, 0.047)	0.040 (0.038, 0.041)	0.041 (0.039, 0.043)	*P* < 0.0001	*P* < 0.0001

All values are non-adjusted mean (95% CIs). Values shown for quartiles of total dairy product consumption are min and max grams consumed per day. The variable was calculated by dividing cost in pounds (£) per day by nutrient intakes per day with a cut-off when 100% of the RNI was met for each nutrient

^a^Based on Bonferroni post hoc test comparing the highest (Q4) and lowest (Q1) dairy quartiles

^a^Differences between dietary cost per mg or µg nutrient across dairy quartiles using general linear models adjusted for age, sex and total energy intake

### Associations between food groups and GHGEs

The association between the average UK diet and diets containing varying quantities of dairy intake with GHGEs was further explored in general linear models that examined the contribution of 15 individual food groups to GHGEs, adjusted for age, sex and energy intake (Supplemental Table 4). The food groups contributing most to GHGEs in the total population were meat and meat products, vegetables and potatoes, dairy products, cereals and cereal products and alcohol contributing 24, 16, 15, 14 and 11%, respectively, of total GHGEs.

Food groups were differentially associated with GHGEs across dairy quartiles, with two food groups (dairy products, fruit) significantly (dairy products *P-*trend < 0.0001; fruit = 0.002) contributing to GHGEs in Q4 compared with Q1 (all Q4 vs. Q1 dairy products *P* < 0.0001; fruit = 0.001). In addition, three food groups (meat and meat products, alcohol, non-alcoholic beverages) were associated with significantly lower (all *P-*trend < 0.0001) contribution to GHGEs in Q4 compared with Q1 (Q4 vs. Q1 all *P* < 0.0001). Among the 15 food groups, the food group contributing most to GHGEs was dairy products, in which diets containing the highest amount of dairy (Q4) had 0.88 kg CO_2_ eqv/day (376%) greater GHGEs than diets containing the least amount of dairy (Q1).

### Associations between food groups and nutrient intakes

The relationship between nutrients and food groups that were significantly different (*P* < 0.0001) across quartiles of total dairy intake is shown in Supplemental Tables 5–17. Food groups were differentially associated with nutrient intakes across increasing quartiles of dairy intake, with cereal and cereal products significantly contributing to intakes of energy (*P*-trend = 0.001), carbohydrate (*P*-trend = 0.0001), zinc (*P*-trend = 0.001), magnesium (*P*-trend = 0.0001), thiamine (*P*-trend = 0.0001) and folate (*P*-trend = 0.0001) in the higher diary diets (Q4) compared with the lower dairy diets (Q1). Milk and milk products contributed significantly more to intakes of SFA, PUFA, protein, calcium, potassium, iodine, riboflavin and vitamin B_12_ intakes in Q4 compared with Q1 (*P*-trend all =  < 0.0001, Q4 vs. Q1 all *P* =  < 0.0001).

## Discussion

In this study, we investigated the nutritional adequacy, cardiometabolic risk profile, diet financial cost and environmental impact of UK diets containing varying quantities of dairy products. In addition, financial and environmental costs were estimated for each diet to assess overall impact of the different diets, and per unit nutrient supplied to estimate the quality costs of each diet.

Adults consuming between 274 and 1429 g/day dairy had significantly higher intakes of essential micronutrients including calcium, iodine, vitamin B_12_ and riboflavin, supporting previous studies [29–32]. Dairy products were also among the major food groups contributing to the higher intakes of these nutrients in the diets containing the highest quantity of dairy intake (Q4). However, other foods also contribute to the higher nutrient intake in these diets. The higher overall AHEI score of those consuming the higher dairy diets compared with the lower dairy diets suggests that consumption of dairy products is associated with a better overall diet quality. Few studies have investigated diets and diet quality associated with dairy intake in UK populations; however, studies conducted in Australian [33] and American [34] adults have also found higher dairy intake to be associated with better overall diet quality. The diets associated with higher dairy intake in the UK population contained more high-fiber breakfast cereals, vegetables, fruit, tea, coffee and water, and lower intakes of alcohol, chips, and soft drinks (not low calorie) compared with the lower dairy diet. Intakes of these foods are associated with a higher diet quality [35]; however, other components of the higher dairy diets were associated with lower diet quality such as higher intakes of sugar, preserves and sweet spreads. The intake of the particular foods reflects habitual diets in the UK. For example, milk is often consumed with breakfast cereals and in tea and coffee within the UK, which is confirmed by the higher wholegrain, other breakfast cereals and tea and coffee intakes within the higher dairy consumers. Furthermore, tea and coffee are also often drunk with biscuits (cookies) or cakes. The intakes of specific foods would be different if the diets of other countries were considered, and this indicates the importance of studying representative diets within countries.

Diets containing the highest amount of dairy products had significantly higher eutrophication potential compared with diets containing the lowest, although there was no significant difference in GHGEs and acidification potential across all levels of dairy intake. Previous studies have investigated the effects of changing specific aspects of the diet such as reducing meat consumption and replacing this with fruits and vegetables, and determining associated reduction in GHGEs [36–40]. In our study, we found that the eutrophication potential was higher for the higher dairy diets compared with the lower intakes, yet the higher dairy diets met significantly more of the nutrient recommendations and had a better AHEI score. This finding supports previous research which showed that self-selected diets of French adults that were of high nutritional quality were not associated with lower GHGEs [41]. One possible explanation may simply be that dairy foods have a high nutrient density but a relatively high environmental impact per kg basis. However, other foods, notably breakfast cereals, also contributed to the environmental impact within this diet, which highlights the importance of calculating the environmental impact in the context of real habitual diets. It is of note that these diets represent usual UK population eating habits and the diets have not been optimized for nutrient intake, financial costs or environmental impact.

The GHGEs per unit nutrient were significantly lower in the higher dairy diets for a number of micronutrients, particularly calcium, iodine, vitamin B_12_ and riboflavin, despite the overall environmental impact of the higher-dairy diets being significantly higher than the lower-dairy diets. Therefore, although diets that contain between 274 and 1429 g/day dairy products had a higher overall environmental impact, these diets are a more efficient and effective way of delivering the required nutrients, which have a relatively lower environmental cost for a higher dietary quality.

The monetary cost of food is an important factor in food choice [42, 43]. In our study, the cost of the average UK adult diet was similar to other studies [28, 37]. We also found that diets containing the highest amount of dairy were cheaper than the average UK diet and the diets containing the lowest amount of dairy. In addition, the financial cost per unit nutrient was significantly lower for a number of nutrients, particularly calcium, iodine, vitamin B_12_ and riboflavin, in the higher compared with the lower quartiles of dairy intake. This may be due to the high concentration of these nutrients in dairy products as well as the lower cost of dairy foods as a source of these nutrients compared with other food groups in the NDNS.

Assessment of the metabolic profile of individuals illustrated that adults in the higher dairy quartile had lower systolic and diastolic blood pressure compared with adults in the lowest quartile of dairy intake. An increasing number of population studies have also shown inverse associations between dairy product consumption and blood pressure, particularly in subjects with hypertension [44, 45]. In addition, Soedamah-Muthu et al. [15] found a lower relative risk of hypertension with higher total dairy in a meta-analysis of nine prospective cohort studies (pooled RR 0.97; 95% CI 0.95, 0.99 per 200 g/day) [15]. This is also supported by intervention studies including the double blind, crossover RCT performed by our group, which reported a significant reduction in 24-h systolic and diastolic blood pressure after consumption of whey protein (56 g/day) for 8 weeks compared with control [46]. One possible mechanism by which dairy product consumption may lower blood pressure is the presence of bioactive peptides, released from milk proteins during digestion, which inhibit the angiotensin I-converting enzyme (ACE) [47].

In our analysis, we found no association between dairy intake and serum TAGs, total-, LDL-C and HDL-C levels, which supports the findings of previous studies which have shown that the fatty acids found within complex dairy foods (excluding butter) have minimal effects on blood lipid concentrations [48]. However, our findings should be interpreted with caution due to the cross-sectional nature of the NDNS study design.

This study faced a number of limitations, including relying on approximate environmental data collected from a number of different sources. There were potential methodological differences, the limited availability of environmental data for every NDNS food group (particularly for acidification and eutrophication potentials) and data were not available for environmental impacts associated with the consumption phase, such as food preparation and waste. Similar considerations apply to the collection of financial data, which were obtained using retail food prices, and therefore only reflect costs at one point in time and only for the foods reported in the NDNS. In addition, the lack of measures of uncertainty in the prices and environmental impacts is another important limitation of this analysis. The cross-sectional design of the NDNS, with no prospective follow-up, is also a limitation. There were quite a few missing samples (*n* = 994) in the health analysis, which means that bias may have incurred. This study is representative of actual dietary intakes in the UK, but may not be representative of the diets of other countries. Despite these limitations, we believe that this study is an important step forward in investigating the environmental impact of typical UK diets using multiple measures of diet-related environmental impact.

## Conclusion

In conclusion, this study, using data from a nationally representative cross-sectional UK population, has shown that diets containing the highest amount of dairy products have higher nutrient intakes, better overall diet quality and lower blood pressure, although are associated with higher eutrophication potential. However, robust data on the environmental costs of many food components were somewhat limited and this requires urgent attention to facilitate determination of the complete picture of the environmental cost of these diets.

## Electronic supplementary material

Below is the link to the electronic supplementary material.

Supplementary material 1 (DOCX 117 kb)

## Abbreviations

ACE: Angiotensin-converting enzyme
AHEI: Alternative Healthy Eating Index
DRV: Dietary reference value
EAR: Estimated average reference
GHGE: Greenhouse gas emissions
LCA: Life-cycle assessment
LDL-C: Low-density lipoprotein cholesterol
HDL-C: High-density lipoprotein cholesterol
NDNS: National diet and nutrition survey
RNI: Reference nutrient intake
