# Supplementary material for: Diets containing the highest levels of dairy products are associated with greater eutrophication potential but higher nutrient intakes and lower financial cost in the United Kingdom
Source: Eur J Nutr. 2019 Mar 29;59(3):895–908. doi: 10.1007/s00394-019-01949-y (PMC7098932; doi:10.1007/s00394-019-01949-y)
Supplement: Supplementary file 1 — Supplementary material 1 (DOCX 117 kb) [file 394_2019_1949_MOESM1_ESM.docx]

**STROBE Statement**

|  | Item No | Recommendation |
| --- | --- | --- |
| **Title and abstract** | 1 | (*a*) Indicate the study’s design with a commonly used term in the title or the abstract **[see line 8, page 2]** |
|  |  | (*b*) Provide in the abstract an informative and balanced summary of what was done and what was found **[see lines 8-21, page 2]** |
| Introduction | | |
| Background/rationale | 2 | Explain the scientific background and rationale for the investigation being reported **[see lines 28-68, pages 4-5]** |
| Objectives | 3 | State specific objectives, including any pre-specified hypotheses  **[see lines 63-68, page 5]** |
| Methods | | |
| Study design | 4 | Present key elements of study design early in the paper [**n/a]** |
| Setting | 5 | Describe the setting, locations, and relevant dates, including periods of recruitment, exposure, follow-up, and data collection [**n/a]** |
| Participants | 6 | (*a*) *Cohort study*—Give the eligibility criteria, and the sources and methods of selection of participants. Describe methods of follow-up  *Case-control study*—Give the eligibility criteria, and the sources and methods of case ascertainment and control selection. Give the rationale for the choice of cases and controls  *Cross-sectional study*—Give the eligibility criteria, and the sources and methods of selection of participants [**n/a]** |
|  |  | (*b*) *Cohort study*—For matched studies, give matching criteria and number of exposed and unexposed  *Case-control study*—For matched studies, give matching criteria and the number of controls per case |
| Variables | 7 | Clearly define all outcomes, exposures, predictors, potential confounders, and effect modifiers. Give diagnostic criteria, if applicable **[see page 6-8]** |
| Data sources/ measurement | 8* | For each variable of interest, give sources of data and details of methods of assessment (measurement). Describe comparability of assessment methods if there is more than one group **[see lines 70-121, pages 6-8]** |
| Bias | 9 | Describe any efforts to address potential sources of bias [**n/a]** |
| Study size | 10 | Explain how the study size was arrived at [**n/a]** |
| Quantitative variables | 11 | Explain how quantitative variables were handled in the analyses. If applicable, describe which groupings were chosen and why **[see lines 123-132, page 8]** |
| Statistical methods | 12 | (*a*) Describe all statistical methods, including those used to control for confounding **[see lines 135-143, page 8]** |
|  |  | (*b*) Describe any methods used to examine subgroups and interactions [**n/a]** |
|  |  | (*c*) Explain how missing data were addressed [**n/a]** |
|  |  | (*d*) *Cohort study*—If applicable, explain how loss to follow-up was addressed  *Case-control study*—If applicable, explain how matching of cases and controls was addressed  *Cross-sectional study*—If applicable, describe analytical methods taking account of sampling strategy [**n/a]** |
|  |  | (*e*) Describe any sensitivity analyses [**n/a]** |

| Results | | |
| --- | --- | --- |
| Participants | 13* | (a) Report numbers of individuals at each stage of study—eg numbers potentially eligible, examined for eligibility, confirmed eligible, included in the study, completing follow-up, and analysed [**n/a]** |
|  |  | (b) Give reasons for non-participation at each stage [**n/a]** |
|  |  | (c) Consider use of a flow diagram [**n/a]** |
| Descriptive data | 14* | (a) Give characteristics of study participants (eg demographic, clinical, social) and information on exposures and potential confounders **[see lines 146-149, page 9 and table 1, page 21]** |
|  |  | (b) Indicate number of participants with missing data for each variable of interest [**n/a]** |
|  |  | (c) *Cohort study*—Summarise follow-up time (eg, average and total amount) |
| Outcome data | 15* | *Cohort study*—Report numbers of outcome events or summary measures over time |
|  |  | *Case-control study—*Report numbers in each exposure category, or summary measures of exposure |
|  |  | *Cross-sectional study—*Report numbers of outcome events or summary measures **[see lines 151-243 and tables 2-7]** |
| Main results | 16 | (*a*) Give unadjusted estimates and, if applicable, confounder-adjusted estimates and their precision (eg, 95% confidence interval). Make clear which confounders were adjusted for and why they were included **[see tables 2-7 and supplemental tables 2-16]** |
|  |  | (*b*) Report category boundaries when continuous variables were categorized **[see lines 123-132, page 8]** |
|  |  | (*c*) If relevant, consider translating estimates of relative risk into absolute risk for a meaningful time period [**n/a]** |
| Other analyses | 17 | Report other analyses done—eg analyses of subgroups and interactions, and sensitivity analyses [**n/a]** |
| Discussion | | |
| Key results | 18 | Summarise key results with reference to study objectives **[see lines 245-250, page]** |
| Limitations | 19 | Discuss limitations of the study, taking into account sources of potential bias or imprecision. Discuss both direction and magnitude of any potential bias **[see lines 324-336, page 16]** |
| Interpretation | 20 | Give a cautious overall interpretation of results considering objectives, limitations, multiplicity of analyses, results from similar studies, and other relevant evidence **[see lines 337-342, page 16]** |
| Generalisability | 21 | Discuss the generalisability (external validity) of the study results **[see lines 337-342, page 16]** |
| Other information | | |
| Funding | 22 | Give the source of funding and the role of the funders for the present study and, if applicable, for the original study on which the present article is based **[please see title page]** |

**Supplemental Figure 1: Participant flow chart**

Numbers of participants aged 19-64 years old recruited, complete data at various stages in years 1-4 of the National Diet and Nutrition Survey (NDNS) and the number of participants included in the final data analysis.

**Original cohort of NDNS (2008/2009-2011/2012) aged 19-64 y** (all with 3-4 d food diary, sex and demographic data)

*n* 1655

**Total included in biomarkers of health analysis**

*n* 661

**Complete biochemical data**

Total cholesterol *n* 808

HDL cholesterol *n* 808

LDL cholesterol *n* 788

Total C-HDL C ratio *n* 808

TAG *n* 806

CRP *n* 809

Glucose *n* 754

HbA1c *n* 799

**Complete blood pressure data**

*n* 1535

**Total included in analysis**

*n* 1655

**Complete anthropometric data**

BMI: *n* 1535

Waist-Hip ratio: *n* 1219

**Complete diet data**

*n* 1655

**The Alternative Healthy Eating Index (AHEI)-2010**

The AHEI-2010 is made up of 11 components, six of which focus on adequacy of the diet (vegetables, fruits, whole grains, nuts and legumes, long-chain omega-3 fatty acids, and poly- unsaturated fatty acids) and the remaining focus on moderation (sugar-sweetened beverages and fruit juices, red and processed meat, *trans* fatty acids, sodium, and alcohol)^(1)^. For the adequacy components, a higher score represented higher consumption. For alcohol, a higher score was given to moderate intake. For the remaining four components (red and processed meats, *trans* fat, sugar- sweetened beverages and fruit juice, and sodium), higher scores represented lower consumption. The sum of component scores resulted in an overall score ranging from 0 (minimal adherence) to 110 (maximal adherence), with a higher total AHEI score indicating a better overall diet quality. More details about the components of the diet scores are shown in **Supplemental Table 1**.

**References**

Chiuve SE1, Fung TT, Rimm EB, Hu FB, McCullough ML, Wang M, Stampfer MJ, Willett WC. Alternative dietary indices both strongly predict risk of chronic disease. J Nutr 2012;142(6):1009-18.

**Environmental methods and data limitations**

*Greenhouse gas emission data*

A Life Cycle Analysis (LCA) approach was used to derive GHGEs from production through to point of retail of the various food groups classified in the NDNS^(1)^. Data used in this study have been obtained from a variety of sources. Based on the data available the LCA represented is up until the point of retail. Data are cross-referenced with at least one other source to ensure they are representative. For some processed foods where a value was not available, the GHGEs have been estimated using the component ingredients of the product. All data sources are either UK specific or provide a reasonable UK proxy, for example the fruit juice figure is based on a European study with transport added which is a representative proxy given that most fruit juice is imported to the UK^(2)^.

The major constraint of these data is that they represent carbon footprints at just one point in time and with on-going changes in technology and practices, figures are subject to change. This is particularly the case where data are from retailers who have carried out research into carbon footprints to provide a baseline for improvement. Additionally, not all data are from the same source, so there may be methodological differences in how carbon dioxide equivalents (CO_2_e) are calculated, although the verification of data sources with secondary data and reliance on LCA data should limit such variation. Selecting a single value to represent an NDNS food group also presents a constraint, given the variety of foods, which are represented within each group. The figure used is based on assumptions relating to the foods within the whole group. Generally, the selection reflects data availability from NDNS or an average of foods within that group. For example, in the juice category, orange juice is the most commonly consumed product so this value was used.

*Biodiversity Metrics*

There is no single quantifiable measure of the impact of food production on biodiversity. We therefore focused on eutrophication potential and acidification potential, where quantifiable information was available.

Eutrophication potential (EP) is defined as the potential of nutrients to cause over-fertilisation of water and soil, which in turn can result in increased growth of biomass. EP data were available by broad food group (e.g. for dairy products, fish etc.) and initial figures were extracted from a collated report of EPs of various foods from databases including Ecoinvent^(3)^. These data were then cross-referenced with additional sources, including values for more specific food groups required for the NDNS food groupings. Data used for some food groups were not always in the standardised units selected (g N/kg food) so conversions from phosphate (PO_4_) and nitrogen dioxide (NO_2_) have been carried out using factors from a EU source (EU, 2006 http://ec.europa.eu/environment/waste/pdf/study/annex5.pdf). For processed NDNS food groups and NDNS meals, where no single value was available, values were calculated using assumed proportions of food ingredients in a meal. This approach also makes the assumption that EP impacts of processing food from ingredients to finished product are minimal. Values collected were not all UK specific and it has been assumed that food produced elsewhere had a similar EP to food produced for the UK market.

Acidification potential (AP) is caused by direct outlets of acids, notably sulphur dioxide (SO_2_), nitrogen oxides (NOx), ammonia (NH_3_) or by outlets of gases that form acid in contact with air humidity and are deposited to soil and water with negative effects on the environment. AP data were sourced in a similar way to EP figures and have the same constraints of only being available for a limited number of food groups. Whilst these have been cross-referenced where possible, this is not always feasible. The same methodology as for EP was used to extrapolate from values for a limited number of broad food groups to values for each NDNS category. The data were not always UK specific and we have made the assumption that processing has a limited influence on the final value. The primary constraint of the biodiversity data is the lack of one single indicator, which accounts for all impacts of food production on biodiversity.

Of the indicators chosen (EP and AP) the main limitation is a lack of data values, which means calculation is involved in assigning values to NDNS groups and processing cannot be accounted for, which may be an issue for EP and AP. Additionally, a lack of data means there is less opportunity to validate values with secondary sources. The values selected are not always UK specific due to the limited sources of data available. As with GHGEs, there are limitations in only accounting for impacts up to a certain stage (farm gate) and not accounting for consumer food preparation and waste.

**References**

1. Bates B, Lennox A, Prentice A, Bates C, Page P, Nicholson S, Swan G. National Diet and Nutrition Survey: headline results from years 1 and 4 combined of the rolling programme 2008/2009–2011/12). Department of Health. Version current 1 2014. Internet: <Https://www.gov.uk/government/uploads/system/uploads/attachment_data/file/310995/NDNS_Y1_to_4_UK_report.pdf> (accessed 24 November 2015).
2. Beccali M, Cellura M, Iudicello M, Mistretta M. Life cycle assessment of Italian citrus-based products. Sensitivity analysis and improvement scenarios. J Environ Manage 2010;91:1415-28.
3. Xue X, Landis AE. Eutrophication potential of food consumption patterns. Environ Sci Technol. 2010;44:6450-6.

**Supplemental Table 1.** Summary of GHGE data and references for each sub-food group

| **Sub-food group** | **kg CO2 eqv/kg food** |
| --- | --- |
| 1% Fat Milk | 1.51 |
| Alcoholic Soft Drinks | 1.80 |
| Apples And Pears Not Canned | 0.40 |
| Baked Beans | 1.82 |
| Bananas | 0.60 |
| Beans And Pulses Incl Ready Meal & Homemade Dishes | 3.30 |
| Beers And Lagers | 1.40 |
| Beverages Dry Weight | 4.50 |
| Biscuits Homemade | 4.47 |
| Biscuits Manufactured / Retail | 4.47 |
| Block Margarine | 2.43 |
| Bottled Water Still Or Carbonated | 0.20 |
| Brown Granary And Wheatgerm Bread | 1.40 |
| Buns Cakes & Pastries Homemade | 4.41 |
| Buns Cakes & Pastries Manufactured | 4.41 |
| Burgers And Kebabs Purchased | 10.10 |
| Butter | 11.08 |
| Canned Fruit In Juice | 1.00 |
| Canned Fruit In Syrup | 0.90 |
| Carrots Not Raw | 1.50 |
| Carrots Raw | 0.70 |
| Cereal Based Milk Puddings - Homemade | 3.33 |
| Cereal Based Milk Puddings - Manufactured | 3.33 |
| Cheddar Cheese | 13.70 |
| Chips Purchased Including Takeaway | 2.00 |
| Chocolate Confectionery | 4.50 |
| Cider And Perry | 1.40 |
| Citrus Fruit Not Canned | 0.90 |
| Coffee (Dry Weight) | 1.91 |
| Cottage Cheese | 13.70 |
| Cream (Including Imitation Cream) | 6.00 |
| Crisps And Savoury Snacks | 3.20 |
| Dairy Desserts Homemade | 3.33 |
| Fortified Wine | 2.40 |
| Fromage Frais And Dairy Desserts Manufactured | 3.90 |
| Fruit Juice | 1.60 |
| Fruit Pies Homemade | 4.08 |
| Fruit Pies Manufactured | 4.08 |
| Green Beans Not Raw | 1.40 |
| Herbal Tea (Dry Weight) | 0.12 |
| High Fibre Breakfast Cereals High Fibre Br'Fast Cereals | 2.10 |
| Homemade Meat Pies And Pastries | 4.00 |
| Ice Cream | 2.40 |
| Leafy Green Vegetables Not Raw | 1.10 |
| Liqueurs | 3.20 |
| Liver And Dishes | 9.6 |
| Low Alcohol & Alcohol Free Beer & Lager | 1.40 |
| Low Alcohol & Alcohol Free Cider & Perry | 1.40 |
| Low Alcohol And Alcohol Free Wine | 2.40 |
| Low Fat Spread Not Polyunsaturated | 2.43 |
| Manufactured Beef Products Including Ready Meals | 12.50 |
| Manufactured Canned Tuna Products Incl Ready Meals | 3.50 |
| Manufactured Chicken Products Incl Ready Meals | 5.10 |
| Manufactured Coated Chicken / Turkey Products | 5.10 |
| Manufactured Egg Products Including Ready Meals | 4.30 |
| Manufactured Lamb Products Including Ready Meals | 10.10 |
| Manufactured Meat Pies And Pastries | 4.00 |
| Manufactured Oily Fish Products Incl Ready Meals | 0.50 |
| Manufactured Pork Products Including Ready Meals | 4.90 |
| Manufactured Shellfish Products Incl Ready Meals | 3.90 |
| Manufactured White Fish Products Incl Ready Meals | 4.10 |
| Meat Alternatives Incl Ready Meals & Homemade Dish | 4.80 |
| Nuts And Seeds | 4.60 |
| Other Bacon And Ham Including Homemade Dishes | 8.20 |
| Other Beef & Veal Including Homemade Recipe Dishes | 12.50 |
| Other Bread | 1.40 |
| Other Breakfast Cereals (Not High Fibre) | 3.00 |
| Other Canned Tuna Including Homemade Dishes | 3.50 |
| Other Cereal Based Puddings - Homemade | 3.33 |
| Other Cereal Based Puddings - Manufactured | 3.33 |
| Other Cereals | 1.30 |
| Other Cheese | 13.70 |
| Other Chicken / Turkey Incl Homemade Recipe Dishes | 5.10 |
| Other Cooking Fats And Oils Not Pufa | 4.30 |
| Other Eggs And Egg Dishes Including Homemade | 4.30 |
| Other Fried / Roast Potatoes Incl Homemade Dishes | 2.00 |
| Other Fruit Not Canned | 2.90 |
| Other Lamb Including Homemade Recipe Dishes | 10.10 |
| Other Manufactured Potato Products Fried/Baked | 1.90 |
| Other Manufactured Vegetable Products Incl Rm | 2.30 |
| Other Meat  Including Homemade Recipe Dishes | 6.40 |
| Other Meat Products Manufactured  Incl Ready Meals | 7.70 |
| Other Milk | 1.00 |
| Other Oily Fish Including Homemade Dishes | 0.50 |
| Other Pasta Including Homemade Dishes (Cooked Weight) | 2.52 |
| Other Pork  Including Homemade Recipe Dishes | 4.90 |
| Other Potato Products &  Dishes - Manufactured | 1.30 |
| Other Potatoes Including  Homemade Dishes | 2.90 |
| Other Rice Including Homemade Dishes | 3.82 |
| Other Sausages Including Homemade Dishes | 4.90 |
| Other Shellfish Including Homemade Dishes | 3.90 |
| Other Vegetables Including Homemade Dishes | 1.90 |
| Other White Fish Including Homemade Dishes | 4.10 |
| Pasta Manufactured Products & Ready Meals | 2.52 |
| Peas Not Raw | 1.80 |
| Pizza | 4.60 |
| Polyunsaturated Low Fat Spread | 2.43 |
| Polyunsaturated Margarine | 2.43 |
| Polyunsaturated Oils | 4.30 |
| Preserves | 3.30 |
| Ready Meals / Meal Centres Based On Bacon And Ham | 8.20 |
| Ready Meals Based On Sausages | 4.90 |
| Reduced Fat Spread (Not Polyunsaturated) | 2.43 |
| Reduced Fat Spread (Polyunsaturated) | 2.43 |
| Rice Manufactured Products & Ready Meals | 3.82 |
| Salad And Other Raw Vegetables | 3.66 |
| Savoury Sauces Pickles Gravies & Condiments | 2.60 |
| Semi Skimmed Milk | 1.61 |
| Skimmed Milk | 1.41 |
| Smoothies 100% Fruit And/Or Juice | 1.60 |
| Soft Drinks Low Calorie Carbonated | 0.27 |
| Soft Drinks Low Calorie Concentrated | 0.43 |
| Soft Drinks Low Calorie Rtd Still | 0.28 |
| Soft Drinks Not Low Calorie Carbonated | 0.60 |
| Soft Drinks Not Low Calorie Concentrated | 6.40 |
| Soft Drinks Not Low Calorie Rtd Still | 0.90 |
| Soft Margarine Not Polyunsaturated | 2.43 |
| Soup Homemade | 3.11 |
| Soup Manufactured/ Retail | 3.10 |
| Soya Milk (Sweetened) | 1.00 |
| Soya Milk (Unsweetened) | 1.00 |
| Soya Milk (Fortified) | 1.10 |
| Spirits | 3.20 |
| Sponge Puddings - Homemade | 3.33 |
| Sponge Puddings - Manufactured | 3.33 |
| Sugar | 0.90 |
| Sugar Confectionery | 4.50 |
| Sweet Spreads Fillings And Icing | 3.30 |
| Tap Water Only | 0.00 |
| Tea (Made-Up Weight) | 0.12 |
| Tomatoes Not Raw | 1.80 |
| Tomatoes Raw | 3.80 |
| White Bread (Not High Fibre; Not Multiseed Bread) | 1.40 |
| White Fish Coated Or Fried | 2.70 |
| Whole Milk | 1.81 |
| Wholegrain & High Fibre Br'Fast Cereals | 2.10 |
| Wholemeal Bread | 1.40 |
| Wine | 2.40 |
| Yogurt | 3.90 |

Values were obtained from the following sources: Tesco product carbon footprint summary (2012)[1], The greenhouse gas footprint of booths (2012)[2], Audsley et al (2009)[3], Nilsson et al (2010)[4], Nielsen et al (2003)[5], Nilsson et al (2011)[6], Beccali et al (2010)[7], Romeo-Games et al (2011)[8], Doublet and Jungbluth (2010)[9], Kasmaprapruet et al (2009)[10], Hospido et al (2009)[11] and DEFRA (2013)[12].

**References**

1. Tesco product carbon footprint summary (2012) Available at <https://www.tescoplc.com/assets/files/cms/Tesco_Product_Carbon_Footprints_Summary(1).pdf>.

2. The greenhouse gas footprint of booths (2012) available at <https://www.booths.co.uk/wp-content/themes/booths/images/Booths%20GHG%20Report%202012%20Final.pdf>.

3. Audsley E, Brander M, Chatterton J, Murphy-Bokern D, Webster C, Williams A How low can we go? An assessment of greenhouse gas emissions from the UK food system and the scope for reduction by 2050. WWF-UK, 2009. Internet: <http://assets.wwf.org.uk/downloads/how_low_report_1.pdf> (accessed on 5 April 2016).

4. Nilsson, K., Flysjö, A., Davis, J., Sim, S., Unger, N. & Bell, S. (2010) 'Comparative life-cycle assessment of margarine and butter consumed in the UK, Germany and France'. International Journal of Life-cycle assessment 15:916-926. Available at <http://mifu.dk/onewebmedia/Comparative%20life%20cycle%20assessment%20of%20margarine%20and%20butter%20consumed%20in%20the%20UK,%20Germany%20and%20France>.pdf.

5. Nielsen PH, Nielsen AM, Weidman BP, Dalgaard R and Halberg N (2003). LCA food data base. "Lifecycle Assessment of Basic Food" (2000 to 2003) Aarhus University, Denmark. Available at <http://www.lcafood.dk/products/crops/bread.htm>.

6. Nilsson,K., Sund,V. and Florén, B. (2011) The environmental impact of the consumption of sweets, crisps and soft drinks A report for Nordic Council of Ministers, Copenhagen 2011. Available at <http://www.fcrn.org.uk/sites/default/files/sweets-crisps-drinks.pdf>.

7. Beccali M, Cellura M, Iudicello M, Mistretta M (2010) Life cycle assessment of Italian citrus-based products. Sensitivity analysis and improvement scenarios. J Environ Manage 91:1415-1428.

8. Romero-Gámez, M., et al., (2011) 'Environmental impact of screenhouse and open-field cultivation using a life-cycle analysis: the case study of green bean production'. Journal of Cleaner Production.

9. Doublet, G. & Jungbluth, N. 2010. Life-cycle assessment of drinking Darjeeling tea: Conventional and organic Darjeeling tea. ESU-services Ltd. .

10. Kasmaprapruet, S., Paengjuntuek, W., Saikhwan, P. and Phungrassami, H., (2009) 'Life-cycle Assessment of Milled Rice Production: Case Study in Thailand'. European Journal of Scientific Research 30(2):195-203.

11. Hospido, A., Milà i Canals, L., McLaren, S., Truninger, M., Edwards-Jones, G. and Clift, R., (2009) 'The role of seasonality in lettuce consumption: a case study of environmental and social aspects'. International Journal of Life-cycle Assessment 14:381– 391.

12. Defra, 2013. 2013 Guidelines to Defra / DECC's GHG Conversion factors for Company Reporting. Available at <https://www.gov.uk/government/collections/government-conversion-factors-for-company-reporting>.

**Supplemental Table 2.** Summary of food prices for each sub-food group

| **Food sub-group** | **£/g food** |
| --- | --- |
| 1% Fat Milk | 0.001 |
| Alcoholic Soft Drinks | 0.003 |
| Apples And Pears Not Canned | 0.002 |
| Baked Beans | 0.001 |
| Bananas | 0.001 |
| Beans And Pulses Incl Ready Meal & Homemade Dishes | 0.003 |
| Beers And Lagers | 0.004 |
| Beverages Dry Weight | 0.006 |
| Biscuits Homemade | 0.002 |
| Biscuits Manufactured / Retail | 0.005 |
| Block Margarine | 0.003 |
| Bottled Water Still Or Carbonated | 0.000 |
| Brown Granary And Wheatgerm Bread | 0.002 |
| Buns Cakes & Pastries Homemade | 0.002 |
| Buns Cakes & Pastries Manufactured | 0.006 |
| Burgers And Kebabs Purchased | 0.004 |
| Butter | 0.004 |
| Canned Fruit In Juice | 0.003 |
| Canned Fruit In Syrup | 0.002 |
| Carrots Not Raw | 0.001 |
| Carrots Raw | 0.001 |
| Cereal Based Milk Puddings - Homemade | 0.001 |
| Cereal Based Milk Puddings - Manufactured | 0.001 |
| Cheddar Cheese | 0.008 |
| Chips Purchased Including Takeaway | 0.003 |
| Chocolate Confectionery | 0.006 |
| Cider And Perry | 0.003 |
| Citrus Fruit Not Canned | 0.002 |
| Coffee (Made-Up Weight) | 0.008 |
| Cottage Cheese | 0.003 |
| Cream (Including Imitation Cream) | 0.003 |
| Crisps And Savoury Snacks | 0.009 |
| Dairy Desserts Homemade | 0.003 |
| Fortified Wine | 0.008 |
| Fromage Frais And Dairy Desserts Manufactured | 0.002 |
| Fruit Juice | 0.001 |
| Fruit Pies Homemade | 0.002 |
| Fruit Pies Manufactured | 0.004 |
| Green Beans Not Raw | 0.004 |
| Herbal Tea (Made-Up Weight) | 0.015 |
| High Fibre Breakfast Cereals High Fibre Br'Fast Cereals | 0.002 |
| Homemade Meat Pies And Pastries | 0.006 |
| Ice Cream | 0.002 |
| Leafy Green Vegetables Not Raw | 0.001 |
| Liqueurs | 0.001 |
| Liver And Dishes | 0.004 |
| Low Alcohol & Alcohol Free Beer & Lager | 0.002 |
| Low Alcohol & Alcohol Free Cider & Perry | 0.002 |
| Low Alcohol And Alcohol Free Wine | 0.003 |
| Low Fat Spread Not Polyunsaturated | 0.003 |
| Manufactured Beef Products Including Ready Meals | 0.006 |
| Manufactured Canned Tuna Products Incl Ready Meals | 0.007 |
| Manufactured Chicken Products Incl Ready Meals | 0.009 |
| Manufactured Coated Chicken / Turkey Products | 0.005 |
| Manufactured Egg Products Including Ready Meals | 0.005 |
| Manufactured Lamb Products Including Ready Meals | 0.005 |
| Manufactured Meat Pies And Pastries | 0.006 |
| Manufactured Oily Fish Products Incl Ready Meals | 0.010 |
| Manufactured Pork Products Including Ready Meals | 0.001 |
| Manufactured Shellfish Products Incl Ready Meals | 0.006 |
| Manufactured White Fish Products Incl Ready Meals | 0.004 |
| Meat Alternatives Incl Ready Meals & Homemade Dish | 0.004 |
| Nuts And Seeds | 0.007 |
| Other Bacon And Ham Including Homemade Dishes | 0.008 |
| Other Beef & Veal Including Homemade Recipe Dishes | 0.011 |
| Other Bread | 0.004 |
| Other Breakfast Cereals (Not High Fibre) | 0.003 |
| Other Canned Tuna Including Homemade Dishes | 0.004 |
| Other Cereal Based Puddings - Homemade | 0.002 |
| Other Cereal Based Puddings - Manufactured | 0.001 |
| Other Cereals | 0.001 |
| Other Cheese | 0.008 |
| Other Chicken / Turkey Incl Homemade Recipe Dishes | 0.005 |
| Other Cooking Fats And Oils Not Pufa | 0.004 |
| Other Eggs And Egg Dishes Including Homemade | 0.003 |
| Other Fried / Roast Potatoes Incl Homemade Dishes | 0.003 |
| Other Fruit Not Canned | 0.007 |
| Other Lamb Including Homemade Recipe Dishes | 0.007 |
| Other Manufactured Potato Products Fried/Baked | 0.001 |
| Other Manufactured Vegetable Products Incl Rm | 0.006 |
| Other Meat  Including Homemade Recipe Dishes | 0.006 |
| Other Meat Products Manufactured  Incl Ready Meals | 0.004 |
| Other Milk | 0.004 |
| Other Oily Fish Including Homemade Dishes | 0.019 |
| Other Pasta Including Homemade Dishes | 0.003 |
| Other Pork  Including Homemade Recipe Dishes | 0.006 |
| Other Potato Products &  Dishes - Manufactured | 0.003 |
| Other Potatoes Including  Homemade Dishes | 0.002 |
| Other Rice Including Homemade Dishes | 0.002 |
| Other Sausages Including Homemade Dishes | 0.006 |
| Other Shellfish Including Homemade Dishes | 0.016 |
| Other Vegetables Including Homemade Dishes | 0.003 |
| Other White Fish Including Homemade Dishes | 0.003 |
| Pasta Manufactured Products & Ready Meals | 0.004 |
| Peas Not Raw | 0.001 |
| Pizza | 0.006 |
| Polyunsaturated Low Fat Spread | 0.002 |
| Polyunsaturated Margarine | 0.002 |
| Polyunsaturated Oils | 0.001 |
| Preserves | 0.003 |
| Ready Meals / Meal Centres Based On Bacon And Ham | 0.009 |
| Ready Meals Based On Sausages | 0.003 |
| Reduced Fat Spread (Not Polyunsaturated) | 0.002 |
| Reduced Fat Spread (Polyunsaturated) | 0.002 |
| Rice Manufactured Products & Ready Meals | 0.005 |
| Salad And Other Raw Vegetables | 0.003 |
| Savoury Sauces Pickles Gravies & Condiments | 0.007 |
| Semi Skimmed Milk | 0.001 |
| Skimmed Milk | 0.001 |
| Smoothies 100% Fruit And/Or Juice | 0.003 |
| Soft Drinks Low Calorie Carbonated | 0.001 |
| Soft Drinks Low Calorie Concentrated | 0.001 |
| Soft Drinks Low Calorie Rtd Still | 0.001 |
| Soft Drinks Not Low Calorie Carbonated | 0.001 |
| Soft Drinks Not Low Calorie Concentrated | 0.001 |
| Soft Drinks Not Low Calorie Rtd Still | 0.002 |
| Soft Margarine Not Polyunsaturated | 0.002 |
| Soup Homemade | 0.002 |
| Soup Manufactured/ Retail | 0.004 |
| Spirits | 0.016 |
| Sponge Puddings - Homemade | 0.002 |
| Sponge Puddings - Manufactured | 0.007 |
| Sugar | 0.006 |
| Sugar Confectionery | 0.005 |
| Sweet Spreads Fillings And Icing | 0.005 |
| Tap Water Only | 0.001 |
| Tea (Made-Up Weight) | 0.008 |
| Tomatoes Not Raw | 0.003 |
| Tomatoes Raw | 0.002 |
| White Bread (Not High Fibre; Not Multiseed Bread) | 0.002 |
| White Fish Coated Or Fried | 0.008 |
| Whole Milk | 0.001 |
| Wholegrain & High Fibre Br'Fast Cereals | 0.000 |
| Wholemeal Bread | 0.002 |
| Wine | 0.006 |
| Yogurt | 0.002 |

**Supplemental Table 3.** Diet scores and components of the Alternative Healthy Eating Index (AHEI-2010)^1^

|  | Scoring | |  | |
| --- | --- | --- | --- | --- |
| Diet scores and components | Min score | Max score | Foods included |  |
| Vegetables, *servings/day*  Fruit, *servings/day*  Whole grains, *g/day*:  Men  Women  Nuts and legumes, *servings/day*  n-3 fatty acids, *mg/day*  PUFA, *% total energy*  Red and processed meat, *servings/day*  Trans-fatty acids, *% total energy*  Sodium, *mg/day*  Sugar-sweetened beverages, *servings/day*  Alcohol*, drinks/day*  Men  Women | No intake  No intake  No intake  No intake  No intake  No intake  ≤2  ≥1.5  ≥4  Highest decile  ≥1  ≥3.5  ≥2.5 | ≥5  ≥4  90  75  ≥1  250  ≥10  No intake  ≥0.5  Lowest decile  No intake  0.5-2.0  0.5-1.5 | All vegetables, excluding potatoes.  All kinds of whole fruits, excluding 100% fruit juices.  Brown and wholemeal breads, rice, pasta and whole grain breakfast cereals (such as muesli, porridge oats, shredded wheat, sultana Bran or Weetabix, bran flakes or puffed wheat and all bran or wheat bran).  All nuts, baked beans, lentils, beans and vegetable protein such as Quorn, soya, tofu.  Sum of n-3 fatty acid content of all foods in diet diary.  Sum of polyunsaturated fat content of all foods in diet diary.  Includes beef, lamb, pork, burgers and processed red meats such as bacon, ham, tinned meats and sausages.  Sum of trans-fatty acids content of all foods in diet diary.  Sum of sodium content of all foods in diet diary.  Sugar-sweetened beverages, fruit juice, fizzy drinks and squashes.  Sum of the alcoholic drinks containing 5 g pure ethanol. |  |

^1^ Scores for the components of the AHEI-2010 index on a continuous scale ranging from 0 to 10 points. Serving sizes are defined as follows: vegetables, 125 g; fruits, 125 g; whole grains, 15 g; legumes, nuts and vegetable proteins (e.g. tofu, soya), 28.4 g; red and processed meat, 113 g; sugar-sweetened beverages, 225 g; and alcohol (wine 113 g, beer 340 g and liquor 52 g).

**Supplemental Table 4.** GHGEs (kg CO_2_ eqv/d) from major food groups across quartiles of total dairy intake^1^

|  |  | Quartiles of dairy product consumption (g/d) | | | |  |  |  |
| --- | --- | --- | --- | --- | --- | --- | --- | --- |
| Food group | Total  *n*=1655 | 1  (0-96)  *n*=411 | 2  (97-172)  *n*=410 | 3  (97-172)  *n*=410 | 4  (274-1429)  *n*=420 | Diff Q4/Q1,  % | *P*  Q1 vs. Q4^2^ | *P*^3^ |
| Cereals and products | 0.57 (0.56, 0.59) | 0.52 (0.49, 0.56) | 0.55 (0.52, 0.58) | 0.61 (0.57, 0.64) | 0.62 (0.59, 0.66) | 19 | 1.00 | 0.05 |
| Dairy products | 0.60 (0.58, 0.62) | 0.22 (0.20, 0.24) | 0.44 (0.41, 0.46) | 0.67 (0.64, 0.69) | 1.1 (1.0, 1.1) | 376 | 0.0001 | 0.0001 |
| Eggs and dishes | 0.081 (0.075, 0.088) | 0.085 (0.064, 0.11) | 0.075 (0.065, 0.085) | 0.082 (0.072, 0.092) | 0.084 (0.073, 0.095) | -1 | 0.09 | 0.11 |
| Fat, spreads and oils | 0.064 (0.060, 0.068) | 0.061 (0.053, 0.070) | 0.059 (0.052, 0.066) | 0.063 (0.057, 0.069) | 0.072 (0.064, 0.081) | 18 | 0.61 | 0.30 |
| Meat and products | 0.99 (0.96, 1.0) | 1.0 (0.97, 1.1) | 0.99 (0.92, 1.1) | 0.93 (0.87, 1.0) | 1.0 (0.94, 1.1) | -4 | 0.0001 | 0.0001 |
| Fish and dishes | 0.078 (0.073, 0.084) | 0.080 (0.068, 0.092) | 0.080 (0.069, 0.091) | 0.076 (0.066, 0.085) | 0.077 (0.066, 0.087) | -4 | 0.37 | 0.22 |
| Vegetables and potatoes | 0.64 (0.63, 0.66) | 0.59 (0.55, 0.62) | 0.63 (0.60, 0.67) | 0.65 (0.61, 0.68) | 0.71 (0.67, 0.74) | 21 | 0.66 | 0.42 |
| Chips | 0.035 (0.033, 0.038) | 0.040 (0.035, 0.046) | 0.035 (0.029, 0.041) | 0.032 (0.027, 0.037) | 0.034 (0.029, 0.039) | -16 | 0.25 | 0.18 |
| Savoury snacks | 0.023 (0.022, 0.025) | 0.024 (0.020, 0.027) | 0.023 (0.020, 0.027) | 0.024 (0.021, 0.027) | 0.022 (0.019, 0.025) | -8 | 0.31 | 0.10 |
| Nuts and seeds | 0.015 (0.013, 0.017) | 0.011 (0.007, 0.014) | 0.017 (0.012, 0.022) | 0.014 (0.010, 0.018) | 0.019 (0.014, 0.023) | 72 | 1.00 | 0.35 |
| Fruit | 0.12 (0.11, 0.13) | 0.080 (0.070, 0.10) | 0.12 (0.10, 0.13) | 0.12 (0.11, 0.13) | 0.16 (0.14, 0.18) | 92 | 0.001 | 0.002 |
| Sugar, preserves, confectionery | 0.064 (0.060, 0.069) | 0.052 (0.045, 0.059) | 0.063 (0.055, 0.071) | 0.068 (0.059, 0.077) | 0.074 (0.065, 0.083) | 42 | 1.00 | 0.38 |
| Non-alcoholic beverages | 0.23 (0.22, 0.25) | 0.26 (0.23, 0.29) | 0.23 (0.20, 0.26) | 0.23 (0.20, 0.26) | 0.21 (0.19, 0.24) | -19 | 0.0001 | 0.0001 |
| Alcoholic beverages | 0.45 (0.41, 0.49) | 0.51 (0.44, 0.59) | 0.50 (0.42, 0.59) | 0.40 (0.33, 0.46) | 0.39 (0.31, 0.46) | -25 | 0.0001 | 0.0001 |
| Miscellaneous | 0.091 (0.086, 0.095) | 0.091 (0.081, 0.10) | 0.084 (0.076, 0.093) | 0.094 (0.085, 0.104) | 0.093 (0.084, 0.102) | 2 | 0.65 | 0.31 |

^1^All values are mean (95% CIs). Values shown for quartiles of total dairy product consumption are min and max grams consumed per day. Data from the National Diet and Nutrition Survey (NDNS) years 1-4 (2008/2009-2011/2012). GHGEs, greenhouse gas emissions; Q, quartile. ^2^Based on Bonferroni post-hoc test comparing the highest and lowest dairy quartiles. ^3^Differences between GHGEs from main food groups across dairy quartiles using general linear models adjusted for age, sex and total energy intake.

**Supplemental Table 5.** Energy intake (kJ/d) from main food groups across quartiles of total dairy intake^1^

|  |  | Quartiles of dairy product consumption (g/d) | | | |  |  |  |
| --- | --- | --- | --- | --- | --- | --- | --- | --- |
|  |  |  |  |  |  |  |  |  |
| Food group | Total  *n*=1655 | 1  (0-96)  *n*=411 | 2  (97-172)  *n*=410 | 3  (97-172)  *n*=410 | 4  (274-1429)  *n*=420 | Diff Q4/Q1, % | *P*  Q1 vs. Q4^2^ | *P*^3^ |
| Cereals and cereal products | 2297 (2245, 2348) | 2017 (1917, 2117) | 2171 (2078, 2263) | 2403 (2304, 2502) | 2589 (2479, 2698) | 28 | 0.048 | 0.001 |
| Milk and milk products | 746 (720, 771) | 318 (292, 344) | 536 (507, 565) | 820 (786, 853) | 1295 (1245, 1346) | 307 | 0.0001 | 0.0001 |
| Eggs and dishes | 146 (134, 158) | 149 (115, 183) | 139 (120, 158) | 147 (128, 166) | 150 (129, 171) | 0 | 0.08 | 0.11 |
| Fat spreads and oils | 333 (320, 347) | 323 (294, 351) | 308 (283, 332) | 326 (303, 349) | 376 (345, 407) | 17 | 0.52 | 0.15 |
| Meat and products | 1142 (1105, 1178) | 1213 (1130, 1296) | 1132 (1061, 1202) | 1055 (989, 1121) | 1167 (1096, 1238) | -4 | 0.0001 | 0.0001 |
| Fish and dishes | 225 (211, 239) | 226 (195, 257) | 232 (202, 262) | 219 (193, 246) | 223 (197, 249) | -1 | 0.029 | 0.020 |
| Vegetables and potatoes | 527 (509, 545) | 483 (445, 520) | 507 (472, 542) | 539 (504, 573) | 578 (544, 613) | 20 | 1.00 | 0.75 |
| Chips | 319 (301, 337) | 372 (331, 412) | 302 (267, 337) | 294 (260, 328) | 307 (275, 340) | -17 | 0.0001 | 0.0001 |
| Savoury snacks | 153 (142, 164) | 159 (135, 182) | 154 (131, 178) | 157 (135, 179) | 143 (122, 164) | -10 | 0.18 | 0.069 |
| Nuts and seeds | 71 (61, 81) | 51 (35, 66) | 74 (54, 95) | 71 (50, 93) | 88 (66, 110) | 74 | 1.00 | 0.65 |
| Fruit | 243 (230, 256) | 181 (156, 206) | 225 (201, 249) | 254 (230, 278) | 311 (281, 341) | 72 | 0.0001 | 0.0001 |
| Sugar, preserves, confectionery | 385 (363, 407) | 307 (272, 342) | 377 (334, 420) | 395 (350, 439) | 459 (410, 508) | 49 | 0.39 | 0.14 |
| Non-alcoholic beverages | 297 (278, 315) | 350 (306, 394) | 279 (245, 312) | 294 (256, 333) | 264 (234, 295) | -24 | 0.0001 | 0.0001 |
| Alcohol | 541 (497, 584) | 620 (527, 713) | 600 (507, 692) | 484 (409, 559) | 461 (373, 549) | -26 | 0.0001 | 0.0001 |
| Miscellaneous | 271 (258, 284) | 285 (256, 315) | 252 (228, 275) | 271 (246, 295) | 276 (250, 301) | -3 | 0.06 | 0.05 |

^1^All values are mean (95% CIs). Values shown for quartiles of total dairy product consumption are min and max grams consumed per day. Data from the National Diet and Nutrition Survey (NDNS) years 1-4 (2008/2009-2011/2012). Q, quartile. ^2^Based on Bonferroni post-hoc test comparing the highest and lowest dairy quartiles. ^3^Differences between dietary energy intake from main food groups across dairy quartiles using general linear models adjusted for age, sex and total energy intake.

**Supplemental Table 6.** Carbohydrate intake (g/d) from main food groups across quartiles of total dairy intake^1^

|  |  | Quartiles of dairy product consumption (g/d) | | | |  |  |  |
| --- | --- | --- | --- | --- | --- | --- | --- | --- |
|  |  |  |  |  |  |  |  |  |
| Food group | Total  *n*=1655 | 1  (0-96)  *n*=411 | 2  (97-172)  *n*=410 | 3  (97-172)  *n*=410 | 4  (274-1429)  *n*=420 | Diff Q4/Q1, % | *P*  Q1 vs. Q4^2^ | *P*^3^ |
| Cereals and cereal products | 98 (96, 101) | 87 (82, 91) | 93 (89, 97) | 103 (99, 107) | 111 (106, 115) | 28 | 0.008 | 0.0001 |
| Milk and milk products | 12 (12, 13) | 3.7 (3.3, 4.1) | 7.9 (7.5, 8.3) | 13 (12, 13) | 24 (23, 25) | 553 | 0.0001 | 0.0001 |
| Eggs and dishes | 0.49 (0.38, 0.59) | 0.32 (0.14, 0.50) | 0.43 (0.24, 0.63) | 0.50 (0.31, 0.68) | 0.69 (0.42, 0.96) | 115 | 0.87 | 0.53 |
| Fat spreads and oils | 0.072 (0.065, 0.078) | 0.07 (0.056, 0.084) | 0.058 (0.049, 0.067) | 0.072 (0.06, 0.083) | 0.086 (0.069, 0.103) | 22 | 1.00 | 0.30 |
| Meat and products | 8.4 (7.9, 9.0) | 9.5 (8.3, 11) | 8.4 (7.3, 9.5) | 7.5 (6.5, 8.4) | 8.4 (7.4, 9.4) | -11 | 0.049 | 0.018 |
| Fish and dishes | 1.8 (1.6, 2.0) | 1.7 (1.4, 2.1) | 1.9 (1.5, 2.3) | 1.8 (1.4, 2.1) | 1.9 (1.5, 2.3) | 10 | 1.00 | 0.92 |
| Vegetables and potatoes | 21 (20, 22) | 19 (17, 20) | 20 (18, 21) | 21 (20, 23) | 23 (22, 24) | 21 | 0.60 | 0.34 |
| Chips | 11 (10, 12) | 13 (11, 14) | 11 (9.0, 12) | 10 (9.0, 11) | 11 (10, 12) | -16 | 0.001 | 0.001 |
| Savoury snacks | 4.3 (4, 4.6) | 4.4 (3.8, 5.1) | 4.3 (3.7, 5.0) | 4.5 (3.8, 5.1) | 4.1 (3.5, 4.7) | -7 | 0.48 | 0.15 |
| Nuts and seeds | 0.39 (0.33, 0.46) | 0.29 (0.16, 0.41) | 0.44 (0.30, 0.58) | 0.39 (0.26, 0.52) | 0.46 (0.33, 0.58) | 60 | 1.00 | 0.63 |
| Fruit | 13 (13, 14) | 10 (9.0, 11) | 12 (11, 14) | 14 (13, 16) | 17 (16, 19) | 77 | 0.0001 | 0.0001 |
| Sugar, preserves, confectionery | 18 (17, 19) | 14 (13, 15) | 17 (15, 19) | 18 (16, 20) | 22 (20, 24) | 57 | 0.014 | 0.019 |
| Non-alcoholic beverages | 17 (16, 18) | 20 (18, 23) | 16 (14, 18) | 17 (14, 19) | 15 (13, 17) | -28 | 0.0001 | 0.0001 |
| Alcohol | 6.3 (5.7, 7.0) | 7.2 (5.9, 8.5) | 6.7 (5.4, 8.1) | 5.7 (4.4, 7.0) | 5.6 (4.4, 6.8) | -22 | 0.0001 | 0.0001 |
| Miscellaneous | 6.6 (6.2, 7.0) | 6.8 (5.9, 7.7) | 6.4 (5.7, 7.0) | 6.4 (5.8, 7.0) | 6.9 (6.2, 7.5) | 1 | 0.55 | 0.29 |

^1^All values are mean (95% CIs). Values shown for quartiles of total dairy product consumption are min and max grams consumed per day. Data from the National Diet and Nutrition Survey (NDNS) years 1-4 (2008/2009-2011/2012). Q, quartile. ^2^Based on Bonferroni post-hoc test comparing the highest and lowest dairy quartiles. ^3^Differences between dietary carbohydrate intake from main food groups across dairy quartiles using general linear models adjusted for age, sex and total energy intake.

**Supplemental Table 7.** Saturated fat intake (g/d) from main food groups across quartiles of total dairy intake^1^

|  |  | Quartiles of dairy product consumption (g/d) | | | |  |  |  |
| --- | --- | --- | --- | --- | --- | --- | --- | --- |
|  |  |  |  |  |  |  |  |  |
| Food group | Total  *n*=1655 | 1  (0-96)  *n*=411 | 2  (97-172)  *n*=410 | 3  (97-172)  *n*=410 | 4  (274-1429)  *n*=420 | Diff Q4/Q1, % | *P*  Q1 vs. Q4^2^ | *P*^3^ |
| Cereals and cereal products | 4.5 (4.3, 4.7) | 3.9 (3.6, 4.2) | 4.2 (3.9, 4.6) | 4.8 (4.4, 5.2) | 5.1 (4.7, 5.5) | 30 | 1.00 | 0.25 |
| Milk and milk products | 6.3 (6.0, 6.5) | 3.1 (2.8, 3.5) | 4.7 (4.3, 5.1) | 7.1 (6.7, 7.6) | 10 (9.4, 10.6) | 219 | 0.0001 | 0.0001 |
| Eggs and dishes | 0.76 (0.69, 0.82) | 0.74 (0.57, 0.92) | 0.74 (0.63, 0.86) | 0.76 (0.66, 0.87) | 0.77 (0.64, 0.90) | 4 | 0.29 | 0.24 |
| Fat spreads and oils | 3.2 (3.0, 3.4) | 3.1 (2.7, 3.4) | 2.9 (2.6, 3.2) | 3.1 (2.8, 3.4) | 3.7 (3.3, 4.1) | 20 | 0.85 | 0.27 |
| Meat and products | 5.3 (5.1, 5.5) | 5.6 (5.2, 6.0) | 5.3 (4.9, 5.6) | 4.9 (4.6, 5.3) | 5.5 (5.1, 5.9) | -2 | 0.0001 | 0.0001 |
| Fish and dishes | 0.60 (0.54, 0.65) | 0.61 (0.49, 0.73) | 0.60 (0.50, 0.71) | 0.58 (0.49, 0.68) | 0.58 (0.49, 0.67) | -5 | 0.057 | 0.057 |
| Vegetables and potatoes | 0.60 (0.56, 0.63) | 0.56 (0.48, 0.64) | 0.59 (0.52, 0.67) | 0.57 (0.50, 0.65) | 0.66 (0.58, 0.74) | 18 | 1.00 | 0.81 |
| Chips | 0.65 (0.61, 0.69) | 0.80 (0.70, 0.90) | 0.60 (0.52, 0.69) | 0.59 (0.51, 0.67) | 0.61 (0.53, 0.68) | -24 | 0.0001 | 0.0001 |
| Savoury snacks | 0.26 (0.24, 0.29) | 0.25 (0.21, 0.29) | 0.28 (0.22, 0.33) | 0.27 (0.22, 0.32) | 0.26 (0.20, 0.32) | 2 | 1.00 | 0.26 |
| Nuts and seeds | 0.33 (0.28, 0.38) | 0.24 (0.15, 0.32) | 0.36 (0.24, 0.48) | 0.31 (0.21, 0.42) | 0.40 (0.29, 0.50) | 66 | 1.00 | 0.54 |
| Fruit | 0.096 (0.081, 0.11) | 0.091 (0.053, 0.129) | 0.11 (0.067, 0.14) | 0.077 (0.063, 0.091) | 0.11 (0.088, 0.14) | 23 | 1.00 | 0.36 |
| Sugar, preserves, confectionery | 1.3 (1.2, 1.4) | 1.1 (0.90, 1.2) | 1.3 (1.1, 1.6) | 1.3 (1.1, 1.5) | 1.4 (1.2, 1.7) | 34 | 1.00 | 0.21 |
| Non-alcoholic beverages | 0.18 (0.15, 0.21) | 0.15 (0.080, 0.22) | 0.14 (0.089, 0.19) | 0.22 (0.15, 0.29) | 0.21 (0.15, 0.27) | 42 | 1.00 | 0.40 |
| Alcohol | 0.035 (0.0080, 0.061) | 0.03 (0.02, 0.08) | 0.04 (0.017, 0.097) | 0.031 (0.005, 0.066) | 0.039 (0.027, 0.10) | 30 | 1.00 | 0.97 |
| Miscellaneous | 0.83 (0.77, 0.88) | 0.85 (0.74, 0.95) | 0.77 (0.66, 0.88) | 0.85 (0.74, 0.95) | 0.84 (0.73, 0.94) | -1 | 0.47 | 0.34 |

^1^All values are mean (95% CIs). Values shown for quartiles of total dairy product consumption are min and max grams consumed per day. Data from the National Diet and Nutrition Survey (NDNS) years 1-4 (2008/2009-2011/2012). Q, quartile. ^2^Based on Bonferroni post-hoc test comparing the highest and lowest dairy quartiles. ^3^Differences between dietary saturated fat intake from main food groups across dairy quartiles using general linear models adjusted for age, sex and total energy intake.

**Supplemental Table 8.** Polyunsaturated fatty acid (PUFA) intake (g/d) from main food groups across quartiles of total dairy intake^1^

|  |  | Quartiles of dairy product consumption (g/d) | | | |  |  |  |
| --- | --- | --- | --- | --- | --- | --- | --- | --- |
|  |  |  |  |  |  |  |  |  |
| Food group | Total  *n*=1655 | 1  (0-96)  *n*=411 | 2  (97-172)  *n*=410 | 3  (97-172)  *n*=410 | 4  (274-1429)  *n*=420 | Diff Q4/Q1, % | *P*  Q1 vs. Q4^2^ | *P*^3^ |
| Cereals and cereal products | 2.3 (2.3, 2.4) | 2.0 (1.9, 2.1) | 2.2 (2.1, 2.4) | 2.4 (2.3, 2.6) | 2.8 (2.6, 2.9) | 38 | 0.22 | 0.15 |
| Milk and milk products | 0.31 (0.30, 0.33) | 0.20 (0.16, 0.24) | 0.24 (0.21, 0.27) | 0.32 (0.30, 0.35) | 0.49 (0.45, 0.52) | 143 | 0.0001 | 0.0001 |
| Eggs and dishes | 0.40 (0.37, 0.44) | 0.43 (0.33, 0.52) | 0.40 (0.34, 0.46) | 0.41 (0.35, 0.46) | 0.39 (0.33, 0.45) | -9 | 0.010 | 0.016 |
| Fat spreads and oils | 1.7 (1.6, 1.8) | 1.7 (1.5, 1.9) | 1.6 (1.5, 1.8) | 1.6 (1.5, 1.8) | 1.9 (1.7, 2.1) | 10 | 1.00 | 0.21 |
| Meat and products | 2.0 (1.9, 2.1) | 2.2 (2.0, 2.3) | 2.0 (1.8, 2.1) | 1.8 (1.7, 2.0) | 2.0 (1.8, 2.1) | -8 | 0.0001 | 0.0001 |
| Fish and dishes | 0.72 (0.66, 0.77) | 0.75 (0.62, 0.88) | 0.76 (0.65, 0.88) | 0.67 (0.57, 0.77) | 0.68 (0.59, 0.78) | -9 | 0.001 | 0.001 |
| Vegetables and potatoes | 1.0 (0.98, 1.1) | 1.0 (0.88, 1.2) | 1.0 (0.89, 1.1) | 1.0 (0.93, 1.2) | 1.1 (0.98, 1.2) | 6 | 0.59 | 0.41 |
| Chips | 0.97 (0.9, 1.0) | 1.1 (0.97, 1.3) | 0.94 (0.81, 1.1) | 0.9 (0.77, 10) | 0.91 (0.8, 1.0) | -18 | 0.001 | 0.001 |
| Savoury snacks | 0.42 (0.37, 0.46) | 0.42 (0.33, 0.52) | 0.45 (0.35, 0.54) | 0.42 (0.35, 0.49) | 0.37 (0.30, 0.45) | -11 | 0.45 | 0.10 |
| Nuts and seeds | 0.45 (0.38, 0.51) | 0.30 (0.20, 0.40) | 0.45 (0.33, 0.57) | 0.45 (0.31, 0.59) | 0.58 (0.42, 0.73) | 92 | 1.00 | 0.53 |
| Fruit | 0.085 (0.077, 0.093) | 0.074 (0.052, 0.096) | 0.083 (0.069, 0.097) | 0.079 (0.069, 0.088) | 0.10 (0.088, 0.12) | 39 | 1.00 | 0.50 |
| Sugar, preserves, confectionery | 0.17 (0.15, 0.19) | 0.16 (0.11, 0.20) | 0.16 (0.13, 0.19) | 0.18 (0.13, 0.24) | 0.17 (0.13, 0.21) | 7 | 1.00 | 0.31 |
| Non-alcoholic beverages | 0.031 (0.027, 0.035) | 0.033 (0.021, 0.045) | 0.029 (0.024, 0.034) | 0.030 (0.025, 0.036) | 0.032 (0.027, 0.038) | -1 | 1.00 | 0.58 |
| Alcohol | - | - | - | - | - | - | - | - |
| Miscellaneous | 1.1 (1.0, 1.2) | 1.1 (1.0, 1.3) | 1.0 (0.9, 1.2) | 1.2 (1, 1.4) | 1.1 (0.9, 1.2) | -6 | 0.20 | 0.10 |

^1^All values are mean (95% CIs). Values shown for quartiles of total dairy product consumption are min and max grams consumed per day. Data from the National Diet and Nutrition Survey (NDNS) years 1-4 (2008/2009-2011/2012). Q, quartile. ^2^Based on Bonferroni post-hoc test comparing the highest and lowest dairy quartiles. ^3^Differences between dietary PUFA intake from main food groups across dairy quartiles using general linear models adjusted for age, sex and total energy intake.

**Supplemental Table 9.** Protein intake (g/d) from main food groups across quartiles of total dairy intake^1^

|  |  | Quartiles of dairy product consumption (g/d) | | | |  |  |  |
| --- | --- | --- | --- | --- | --- | --- | --- | --- |
|  |  |  |  |  |  |  |  |  |
| Food group | Total  *n*=1655 | 1  (0-96)  *n*=411 | 2  (97-172)  *n*=410 | 3  (97-172)  *n*=410 | 4  (274-1429)  *n*=420 | Diff Q4/Q1, % | *P*  Q1 vs. Q4^2^ | *P*^3^ |
| Cereals and cereal products | 16 (16, 16) | 16 (16, 16) | 14 (14, 15) | 15 (14, 16) | 16 (16, 17) | 24 | 0.44 | 0.035 |
| Milk and milk products | 10 (10, 11) | 10 (10, 11) | 4.2 (3.8, 4.5) | 7.6 (7.3, 8.0) | 11 (11, 12) | 343 | 0.0001 | 0.0001 |
| Eggs and dishes | 2.4 (2.2, 2.6) | 2.4 (2.2, 2.6) | 2.5 (1.9, 3.1) | 2.2 (1.9, 2.5) | 2.4 (2.1, 2.7) | -4 | 0.047 | 0.06 |
| Fat spreads and oils | 0.042 (0.039, 0.045) | 0.042 (0.039, 0.045) | 0.038 (0.033, 0.043) | 0.039 (0.033, 0.045) | 0.045 (0.038, 0.051) | 24 | 1.00 | 0.66 |
| Meat and products | 27 (27, 28) | 27 (27, 28) | 29 (27, 31) | 27 (26, 29) | 26 (24, 27) | -2 | 0.0001 | 0.0001 |
| Fish and dishes | 5.6 (5.3, 6.0) | 5.6 (5.3, 6.0) | 5.7 (4.9, 6.5) | 5.7 (5.1, 6.4) | 5.6 (4.9, 6.2) | -2 | 0.049 | 0.045 |
| Vegetables and potatoes | 5.1 (4.9, 5.2) | 5.1 (4.9, 5.2) | 4.4 (4.1, 4.8) | 4.9 (4.5, 5.3) | 5.3 (4.9, 5.7) | 26 | 0.12 | 0.088 |
| Chips | 1.2 (1.1, 1.3) | 1.2 (1.1, 1.3) | 1.4 (1.2, 1.5) | 1.1 (4.2, 1.3) | 1.1 (4.2, 1.2) | -16 | 0.0001 | 0.0001 |
| Savoury snacks | 0.46 (0.43, 0.50) | 0.46 (0.43, 0.50) | 0.48 (0.40, 0.56) | 0.46 (0.39, 0.53) | 0.48 (0.41, 0.55) | -10 | 0.30 | 0.11 |
| Nuts and seeds | 0.59 (0.50, 0.67) | 0.59 (0.50, 0.67) | 0.43 (0.29, 0.57) | 0.61 (0.43, 0.78) | 0.61 (0.41, 0.80) | 62 | 1.00 | 0.79 |
| Fruit | 0.77 (0.73, 0.81) | 0.77 (0.73, 0.81) | 0.56 (0.49, 0.64) | 0.73 (0.65, 0.82) | 0.81 (0.73, 0.89) | 72 | 0.0001 | 0.001 |
| Sugar, preserves, confectionery | 0.64 (0.59, 0.70) | 0.64 (0.59, 0.70) | 0.55 (0.44, 0.66) | 0.66 (0.54, 0.78) | 0.67 (0.55, 0.80) | 26 | 1.00 | 0.18 |
| Non-alcoholic beverages | 0.75 (0.69, 0.82) | 0.75 (0.69, 0.82) | 0.66 (0.51, 0.81) | 0.7 (0.59, 0.81) | 0.81 (0.66, 1.0) | 28 | 1.00 | 0.83 |
| Alcohol | 0.62 (0.55, 0.69) | 0.62 (0.55, 0.69) | 0.72 (0.58, 0.87) | 0.68 (0.54, 0.81) | 0.51 (0.39, 0.63) | -21 | 0.0001 | 0.0001 |
| Miscellaneous | 1.6 (1.5, 1.8) | 1.6 (1.4, 1.8) | 4.2 (1.5, 2.6) | 1.4 (1.2, 1.6) | 1.3 (1.1, 1.5) | -13 | 0.94 | 0.032 |

^1^All values are mean (95% CIs). Values shown for quartiles of total dairy product consumption are min and max grams consumed per day. Data from the National Diet and Nutrition Survey (NDNS) years 1-4 (2008/2009-2011/2012). Q, quartile. ^2^Based on Bonferroni post-hoc test comparing the highest and lowest dairy quartiles. ^3^Differences between dietary protein intake from main food groups across dairy quartiles using general linear models adjusted for age, sex and total energy intake.

**Supplemental Table 10.** Calcium intake (mg/d) from main food groups across quartiles of total dairy intake^1^

|  |  | Quartiles of dairy product consumption (g/d) | | | |  |  |  |
| --- | --- | --- | --- | --- | --- | --- | --- | --- |
|  |  |  |  |  |  |  |  |  |
| Food group | Total  *n*=1655 | 1  (0-96)  *n*=411 | 2  (97-172)  *n*=410 | 3  (97-172)  *n*=410 | 4  (274-1429)  *n*=420 | Diff Q4/Q1, % | *P*  Q1 vs. Q4^2^ | *P*^3^ |
| Cereals and cereal products | 230 (224, 236) | 212 (200, 224) | 223 (212, 234) | 230 (219, 241) | 255 (241, 269) | 20 | 1.00 | 0.87 |
| Milk and milk products | 333 (323, 344) | 127 (117, 136) | 236 (227, 246) | 361 (350, 371) | 604 (584, 623) | 376 | 0.0001 | 0.0001 |
| Eggs and dishes | 13 (12, 14) | 13 (9.0, 16) | 12 (10, 14) | 13 (11, 15) | 13 (11, 15) | 5 | 0.40 | 0.33 |
| Fat spreads and oils | 2.4 (2.3, 2.5) | 2.3 (2, 2.5) | 2.2 (1.9, 2.4) | 2.4 (2.2, 2.6) | 2.8 (2.5, 3.1) | 24 | 1.00 | 0.60 |
| Meat and products | 45 (43, 47) | 48 (44, 53) | 45 (41, 49) | 41 (37, 45) | 46 (42, 51) | -4 | 0.022 | 0.007 |
| Fish and dishes | 19 (17, 21) | 19 (16, 23) | 18 (15, 22) | 18 (15, 21) | 20 (16, 23) | 4 | 0.80 | 0.34 |
| Vegetables and potatoes | 53 (51, 55) | 48 (44, 52) | 52 (48, 55) | 56 (52, 59) | 58 (54, 62) | 21 | 1.00 | 0.46 |
| Chips | 4.4 (4.1, 4.8) | 5.1 (4.4, 5.7) | 4.1 (3.5, 4.6) | 4.3 (3.7, 4.9) | 4.3 (3.7, 5.0) | -14 | 0.029 | 0.035 |
| Savoury snacks | 3.0 (2.7, 3.3) | 2.9 (2.4, 3.4) | 3.2 (2.4, 3.9) | 3.3 (2.8, 3.9) | 2.6 (2.2, 3.0) | -9 | 0.96 | 0.026 |
| Nuts and seeds | 2.7 (2.2, 3.2) | 1.8 (1.1, 2.6) | 3.4 (2.1, 4.7) | 2.2 (1.5, 2.9) | 3.5 (2.4, 4.6) | 90 | 1.00 | 0.16 |
| Fruit | 13 (12, 14) | 9.2 (7.7, 11) | 13 (11, 15) | 14 (12, 16) | 16 (14, 18) | 74 | 0.046 | 0.041 |
| Sugar, preserves, confectionery | 15 (14, 17) | 12 (10, 14) | 16 (13, 19) | 16 (14, 19) | 16 (14, 19) | 33 | 1.00 | 0.086 |
| Non-alcoholic beverages | 27 (25, 29) | 28 (23, 32) | 24 (21, 28) | 29 (24, 33) | 27 (24, 31) | -1 | 1.00 | 0.38 |
| Alcohol | 18 (17, 20) | 20 (17, 24) | 20 (16, 24) | 16 (13, 19) | 16 (13, 19) | -20 | 0.0001 | 0.0001 |
| Miscellaneous | 26 (23, 28) | 31 (23, 39) | 21 (19, 24) | 24 (20, 27) | 28 (23, 33) | -10 | 1.00 | 0.046 |

^1^All values are mean (95% CIs). Values shown for quartiles of total dairy product consumption are min and max grams consumed per day. Data from the National Diet and Nutrition Survey (NDNS) years 1-4 (2008/2009-2011/2012). Q, quartile. ^2^Based on Bonferroni post-hoc test comparing the highest and lowest dairy quartiles. ^3^Differences between dietary calcium intake from main food groups across dairy quartiles using general linear models adjusted for age, sex and total energy intake.

**Supplemental Table 11.** Magnesium intake (mg/d) from main food groups across quartiles of total dairy intake^1^

|  |  | Quartiles of dairy product consumption (g/d) | | | |  |  |  |
| --- | --- | --- | --- | --- | --- | --- | --- | --- |
|  |  |  |  |  |  |  |  |  |
| Food group | Total  *n*=1655 | 1  (0-96)  *n*=411 | 2  (97-172)  *n*=410 | 3  (97-172)  *n*=410 | 4  (274-1429)  *n*=420 | Diff Q4/Q1, % | *P*  Q1 vs. Q4^2^ | *P*^3^ |
| Cereals and cereal products | 68 (66, 70) | 56 (52, 59) | 63 (60, 66) | 72 (69, 75) | 82 (78, 87) | 48 | 0.0001 | 0.0001 |
| Milk and milk products | 26 (25, 27) | 9.0 (8.0, 10) | 18 (17, 18) | 27 (27, 28) | 49 (48, 51) | 447 | 0.0001 | 0.0001 |
| Eggs and dishes | 2.5 (2.3, 2.7) | 2.6 (2.0, 3.1) | 2.3 (2.0, 2.6) | 2.5 (2.2, 2.8) | 2.5 (2.2, 2.9) | -1 | 0.09 | 0.11 |
| Fat spreads and oils | 0.29 (0.27, 0.30) | 0.27 (0.24, 0.3) | 0.27 (0.24, 0.29) | 0.27 (0.25, 0.30) | 0.34 (0.30, 0.37) | 25 | 1.00 | 0.48 |
| Meat and products | 30 (29, 31) | 31 (29, 34) | 30 (28, 32) | 27 (26, 29) | 30 (28, 32) | -5 | 0.0001 | 0.0001 |
| Fish and dishes | 8.4 (7.9, 8.9) | 8.5 (7.4, 9.6) | 8.4 (7.4, 9.5) | 8.3 (7.3, 9.3) | 8.3 (7.3, 9.3) | -2 | 0.042 | 0.045 |
| Vegetables and potatoes | 33 (32, 34) | 30 (28, 32) | 32 (30, 34) | 34 (32, 37) | 37 (34, 39) | 22 | 0.45 | 0.27 |
| Chips | 10 (9.0, 11) | 12 (10, 13) | 9.0 (8.0, 11) | 9.0 (8.0, 10) | 10 (9.0, 11) | -16 | 0.0001 | 0.0001 |
| Savoury snacks | 3.5 (3.2, 3.8) | 3.4 (2.9, 4.0) | 3.7 (3.0, 4.4) | 3.6 (3.1, 4.2) | 3.3 (2.7, 3.8) | -5 | 0.68 | 0.07 |
| Nuts and seeds | 5.7 (4.9, 6.5) | 4 (2.7, 5.3) | 5.9 (4.2, 7.6) | 5.7 (4.0, 7.5) | 7.1 (5.4, 8.8) | 78 | 1.00 | 0.66 |
| Fruit | 15 (14, 16) | 11 (9, 12) | 14 (12, 15) | 16 (14, 17) | 19 (17, 21) | 74 | 0.0001 | 0.0001 |
| Sugar, preserves, confectionery | 4.8 (4.4, 5.3) | 3.9 (3.1, 4.7) | 4.8 (3.9, 5.6) | 5.4 (4.3, 6.4) | 5.3 (4.4, 6.1) | 35 | 1.00 | 0.20 |
| Non-alcoholic beverages | 17 (17, 18) | 15 (14, 16) | 16 (15, 17) | 18 (17, 19) | 20 (19, 21) | 35 | 0.064 | 0.045 |
| Alcohol | 20 (18, 22) | 23 (19, 26) | 22 (18, 25) | 17 (14, 21) | 18 (14, 21) | -21 | 0.0001 | 0.0001 |
| Miscellaneous | 9.3 (8.4, 10.2) | 11.8 (8.6, 15) | 8.3 (7.4, 9.2) | 8.7 (7.7, 9.6) | 8.6 (7.6, 9.5) | -28 | 0.006 | 0.003 |

^1^All values are mean (95% CIs). Values shown for quartiles of total dairy product consumption are min and max grams consumed per day. Data from the National Diet and Nutrition Survey (NDNS) years 1-4 (2008/2009-2011/2012). Q, quartile. ^2^Based on Bonferroni post-hoc test comparing the highest and lowest dairy quartiles. ^3^Differences between dietary magnesium intake from main food groups across dairy quartiles using general linear models adjusted for age, sex and total energy intake.

**Supplemental Table 12.** Potassium intake (mg/d) from major food groups across quartiles of total dairy intake^1^

|  |  | Quartiles of dairy product consumption (g/d) | | | |  |  |  |
| --- | --- | --- | --- | --- | --- | --- | --- | --- |
|  |  |  |  |  |  |  |  |  |
| Food group | Total  *n*=1655 | 1  (0-96)  *n*=411 | 2  (97-172)  *n*=410 | 3  (97-172)  *n*=410 | 4  (274-1429)  *n*=420 | Diff Q4/Q1, % | *P*  Q1 vs. Q4^2^ | *P*^3^ |
| Cereals and cereal products | 364 (354, 373) | 300 (282, 318) | 336 (321, 351) | 384 (367, 401) | 433 (412, 454) | 44 | 0.0001 | 0.0001 |
| Milk and milk products | 329 (317, 341) | 95 (88, 101) | 213 (208, 218) | 345 (338, 351) | 656 (634, 678) | 592 | 0.0001 | 0.0001 |
| Eggs and dishes | 27 (24, 29) | 28 (21, 34) | 24 (21, 28) | 27 (24, 31) | 27 (23, 30) | -4 | 0.040 | 0.056 |
| Fat spreads and oils | 6.8 (6.5, 7.2) | 6.4 (5.6, 7.1) | 6.3 (5.6, 7.0) | 6.5 (5.9, 7.2) | 8.1 (7.2, 9.0) | 26 | 1.00 | 0.47 |
| Meat and products | 379 (367, 392) | 397 (366, 429) | 379 (356, 402) | 354 (333, 375) | 387 (364, 410) | -3 | 0.0001 | 0.0001 |
| Fish and dishes | 91 (85, 97) | 93 (80, 106) | 89 (78, 100) | 92 (81, 103) | 91 (80, 102) | -2 | 0.073 | 0.094 |
| Vegetables and potatoes | 546 (529, 562) | 480 (447, 513) | 524 (491, 557) | 564 (531, 597) | 613 (579, 647) | 28 | 0.042 | 0.046 |
| Chips | 210 (198, 222) | 245 (219, 272) | 199 (175, 222) | 194 (171, 216) | 203 (181, 225) | -17 | 0.0001 | 0.0001 |
| Savoury snacks | 58 (54, 62) | 60 (51, 69) | 57 (49, 66) | 58 (50, 67) | 56 (47, 65) | -6 | 0.52 | 0.30 |
| Nuts and seeds | 21 (18, 24) | 15 (10, 20) | 22 (16, 28) | 21 (15, 28) | 27 (20, 35) | 79 | 1.00 | 0.71 |
| Fruit | 217 (205, 229) | 159 (137, 181) | 200 (178, 221) | 228 (207, 250) | 280 (253, 307) | 76 | 0.0001 | 0.0001 |
| Sugar, preserves, confectionery | 36 (32, 39) | 29 (23, 34) | 36 (29, 42) | 39 (31, 47) | 39 (32, 45) | 35 | 1.00 | 0.21 |
| Non-alcoholic beverages | 249 (239, 260) | 199 (179, 219) | 237 (218, 255) | 265 (244, 285) | 296 (275, 318) | 49 | 0.002 | 0.002 |
| Alcohol | 146 (133, 158) | 162 (136, 188) | 162 (132, 191) | 132 (110, 155) | 127 (103, 150) | -22 | 0.0001 | 0.0001 |
| Miscellaneous | 108 (101, 114) | 118 (103, 132) | 105 (93, 118) | 106 (94, 118) | 102 (90, 113) | -14 | 0.054 | 0.08 |

^1^All values are mean (95% CIs). Values shown for quartiles of total dairy product consumption are min and max grams consumed per day. Data from the National Diet and Nutrition Survey (NDNS) years 1-4 (2008/2009-2011/2012). Q, quartile. ^2^Based on Bonferroni post-hoc test comparing the highest and lowest dairy quartiles. ^3^Differences between dietary potassium intake from main food groups across dairy quartiles using general linear models adjusted for age, sex and total energy intake.

**Supplemental Table 13.** Iodine intake (μg/d) from major food groups across quartiles of total dairy intake^1^

|  |  | Quartiles of dairy product consumption (g/d) | | | |  |  |  |
| --- | --- | --- | --- | --- | --- | --- | --- | --- |
|  |  |  |  |  |  |  |  |  |
| Food group | Total  *n*=1655 | 1  (0-96)  *n*=411 | 2  (97-172)  *n*=410 | 3  (97-172)  *n*=410 | 4  (274-1429)  *n*=420 | Diff Q4/Q1, % | *P*  Q1 vs. Q4^2^ | *P*^3^ |
| Cereals and cereal products | 15 (14, 15) | 12 (11, 13) | 15 (14, 16) | 15 (14, 16) | 17 (16, 18) | 39 | 0.69 | 0.10 |
| Milk and milk products | 57 (55, 59) | 17 (16, 18) | 38 (37, 39) | 61 (59, 63) | 110 (105, 114) | 558 | 0.0001 | 0.0001 |
| Eggs and dishes | 10 (8.7, 10) | 10 (7.6, 13) | 8.8 (7.6, 10) | 9.4 (8.3, 11) | 10 (8.4, 11) | -8 | 0.020 | 0.031 |
| Fat spreads and oils | 3.4 (3.2, 3.7) | 3.4 (2.7, 4.0) | 3.0 (2.7, 3.3) | 3.4 (2.8, 4.0) | 3.9 (3.4, 4.3) | 14 | 1.00 | 0.35 |
| Meat and products | 11 (10, 11) | 11 (10, 12) | 11 (10, 12) | 10 (9, 11) | 11 (10, 12) | -1 | 0.001 | 0.0001 |
| Fish and dishes | 20 (18, 22) | 22 (18, 26) | 19 (16, 23) | 21 (17, 24) | 17 (14, 20) | -22 | 0.004 | 0.009 |
| Vegetables and potatoes | 6.1 (5.9, 6.3) | 5.5 (5.1, 5.9) | 5.8 (5.4, 6.2) | 6.3 (5.9, 6.7) | 6.6 (6.3, 7.0) | 22 | 0.81 | 0.32 |
| Chips | 1.8 (1.7, 1.9) | 2.1 (1.9, 2.3) | 1.7 (1.5, 1.9) | 1.6 (1.4, 1.8) | 1.7 (1.6, 1.9) | -16 | 0.0001 | 0.0001 |
| Savoury snacks | 0.20 (0.18, 0.22) | 0.20 (0.17, 0.23) | 0.19 (0.16, 0.23) | 0.22 (0.18, 0.26) | 0.19 (0.16, 0.22) | -5 | 1.00 | 0.17 |
| Nuts and seeds | 0.31 (0.26, 0.37) | 0.25 (0.15, 0.34) | 0.27 (0.18, 0.36) | 0.32 (0.20, 0.44) | 0.40 (0.29, 0.52) | 63 | 1.00 | 0.96 |
| Fruit | 3.2 (3.1, 3.4) | 2.3 (1.9, 2.6) | 3.0 (2.6, 3.4) | 3.5 (3.1, 3.9) | 4.2 (3.8, 4.6) | 86 | 0.0001 | 0.0001 |
| Sugar, preserves, confectionery | 2.6 (2.4, 2.9) | 2.1 (1.6, 2.7) | 2.7 (2.1, 3.3) | 2.8 (2.4, 3.3) | 2.8 (2.3, 3.3) | 31 | 1.00 | 0.24 |
| Non-alcoholic beverages | 7.3 (6.9, 7.8) | 5.1 (4.1, 6.1) | 6.6 (5.8, 7.3) | 8.8 (7.8, 9.8) | 8.7 (7.9, 9.5) | 71 | 0.002 | 0.0001 |
| Alcohol | 19 (17, 21) | 22 (18, 26) | 21 (18, 25) | 16 (13, 19) | 17 (13, 20) | -24 | 0.0001 | 0.0001 |
| Miscellaneous | 5.8 (5.3, 6.2) | 6.5 (5.1, 7.8) | 5.0 (4.3, 5.7) | 5.2 (4.5, 5.8) | 6.4 (5.4, 7.4) | -1 | 1.00 | 0.07 |

^1^All values are mean (95% CIs). Values shown for quartiles of total dairy product consumption are min and max grams consumed per day. Data from the National Diet and Nutrition Survey (NDNS) years 1-4 (2008/2009-2011/2012). Q, quartile. ^2^Based on Bonferroni post-hoc test comparing the highest and lowest dairy quartiles. ^3^Differences between dietary iodine intake from main food groups across dairy quartiles using general linear models adjusted for age, sex and total energy intake.

**Supplemental Table 14.** Zinc intake (mg/d) from main food groups across quartiles of total dairy intake^1^

|  |  | Quartiles of dairy product consumption (g/d) | | | |  |  |  |
| --- | --- | --- | --- | --- | --- | --- | --- | --- |
|  |  |  |  |  |  |  |  |  |
| Food group | Total  *n*=1655 | 1  (0-96)  *n*=411 | 2  (97-172)  *n*=410 | 3  (97-172)  *n*=410 | 4  (274-1429)  *n*=420 | Diff Q4/Q1, % | *P*  Q1 vs. Q4^2^ | *P*^3^ |
| Cereals and cereal products | 1.4 (1.3, 1.4) | 0.57 (0.52, 0.62) | 1.0 (0.94, 1.1) | 1.5 (1.4, 1.6) | 2.4 (2.3, 2.4) | 33 | 0.001 | 0.001 |
| Milk and milk products | 0.059 (0.049, 0.069) | 0.056 (0.034, 0.077) | 0.057 (0.041, 0.073) | 0.055 (0.040, 0.071) | 0.069 (0.044, 0.093) | 314 | 0.0001 | 0.0001 |
| Eggs and dishes | 0.13 (0.13, 0.14) | 0.10 (0.090, 0.11) | 0.12 (0.11, 0.14) | 0.14 (0.12, 0.15) | 0.17 (0.15, 0.18) | -5 | 0.040 | 0.057 |
| Fat spreads and oils | 0.78 (0.74, 0.81) | 0.66 (0.59, 0.72) | 0.76 (0.69, 0.83) | 0.81 (0.73, 0.89) | 0.87 (0.79, 0.95) | 34 | 1.00 | 0.27 |
| Meat and products | 0.17 (0.16, 0.18) | 0.20 (0.18, 0.22) | 0.16 (0.14, 0.18) | 0.16 (0.14, 0.18) | 0.16 (0.15, 0.18) | 0 | 0.002 | 0.0001 |
| Fish and dishes | 2.0 (1.9, 2.0) | 1.7 (1.6, 1.8) | 1.9 (1.8, 1.9) | 2.1 (2.0, 2.1) | 2.3 (2.2, 2.4) | -21 | 0.003 | 0.004 |
| Vegetables and potatoes | 0.061 (0.053, 0.07) | 0.045 (0.030, 0.061) | 0.052 (0.037, 0.066) | 0.078 (0.057, 0.10) | 0.068 (0.052, 0.084) | 32 | 0.080 | 0.070 |
| Chips | 0.016 (0.014, 0.017) | 0.014 (0.012, 0.017) | 0.016 (0.013, 0.018) | 0.014 (0.012, 0.016) | 0.019 (0.016, 0.023) | -18 | 0.0001 | 0.0001 |
| Savoury snacks | 3.0 (2.9, 3.1) | 3.1 (2.9, 3.3) | 3.0 (2.8, 3.2) | 2.8 (2.6, 3.0) | 3.1 (2.9, 3.3) | -5 | 1.00 | 0.11 |
| Nuts and seeds | 0.25 (0.23, 0.27) | 0.29 (0.22, 0.36) | 0.24 (0.21, 0.27) | 0.23 (0.20, 0.26) | 0.23 (0.20, 0.26) | 74 | 1.00 | 0.59 |
| Fruit | 0.11 (0.10, 0.12) | 0.090 (0.070, 0.11) | 0.11 (0.090, 0.12) | 0.12 (0.10, 0.14) | 0.12 (0.11, 0.14) | 67 | 0.0001 | 0.001 |
| Sugar, preserves, confectionery | 0.24 (0.22, 0.27) | 0.26 (0.19, 0.33) | 0.22 (0.19, 0.25) | 0.25 (0.22, 0.28) | 0.25 (0.22, 0.28) | 35 | 1.00 | 0.57 |
| Non-alcoholic beverages | 0.060 (0.050, 0.060) | 0.050 (0.040, 0.060) | 0.060 (0.050, 0.070) | 0.060 (0.050, 0.070) | 0.050 (0.040, 0.060) | 50 | 1.00 | 0.10 |
| Alcohol | 0.090 (0.080, 0.11) | 0.070 (0.040, 0.090) | 0.10 (0.070, 0.13) | 0.10 (0.070, 0.13) | 0.11 (0.090, 0.14) | 23 | 0.17 | 0.14 |
| Miscellaneous | 0.25 (0.21, 0.29) | 0.36 (0.21, 0.51) | 0.19 (0.16, 0.22) | 0.21 (0.18, 0.24) | 0.24 (0.20, 0.28) | -33 | 0.14 | 0.009 |

^1^All values are mean (95% CIs). Values shown for quartiles of total dairy product consumption are min and max grams consumed per day. Data from the National Diet and Nutrition Survey (NDNS) years 1-4 (2008/2009-2011/2012). Q, quartile. ^2^Based on Bonferroni post-hoc test comparing the highest and lowest dairy quartiles. ^3^Differences between dietary zinc intake from main food groups across dairy quartiles using general linear models adjusted for age, sex and total energy intake.

**Supplemental Table 15.** Thiamin intake (mg/d) from main food groups across quartiles of total dairy intake^1^

|  |  | Quartiles of dairy product consumption (g/d) | | | |  |  |  |
| --- | --- | --- | --- | --- | --- | --- | --- | --- |
|  |  |  |  |  |  |  |  |  |
| Food group | Total  *n*=1655 | 1  (0-96)  *n*=411 | 2  (97-172)  *n*=410 | 3  (97-172)  *n*=410 | 4  (274-1429)  *n*=420 | Diff Q4/Q1, % | *P*  Q1 vs. Q4^2^ | *P*^3^ |
| Cereals and cereal products | 0.48 (0.47, 0.50) | 0.38 (0.36, 0.40) | 0.44 (0.42, 0.46) | 0.52 (0.50, 0.55) | 0.59 (0.56, 0.62) | 58 | 0.0001 | 0.0001 |
| Milk and milk products | 0.093 (0.089, 0.096) | 0.028 (0.025, 0.031) | 0.062 (0.059, 0.066) | 0.098 (0.094, 0.10) | 0.18 (0.17, 0.19) | 540 | 0.0001 | 0.0001 |
| Eggs and dishes | 0.015 (0.014, 0.016) | 0.016 (0.012, 0.020) | 0.014 (0.012, 0.016) | 0.015 (0.013, 0.017) | 0.015 (0.013, 0.017) | -5 | 0.046 | 0.066 |
| Fat spreads and oils | - | - | - | - | - | - | - | - |
| Meat and products | 0.29 (0.28, 0.30) | 0.30 (0.27, 0.32) | 0.28 (0.26, 0.31) | 0.27 (0.25, 0.29) | 0.30 (0.27, 0.33) | 2 | 0.028 | 0.011 |
| Fish and dishes | 0.028 (0.025, 0.030) | 0.027 (0.022, 0.032) | 0.028 (0.023, 0.032) | 0.027 (0.023, 0.031) | 0.028 (0.024, 0.033) | 5 | 0.23 | 0.17 |
| Vegetables and potatoes | 0.26 (0.25, 0.26) | 0.22 (0.20, 0.24) | 0.25 (0.23, 0.26) | 0.26 (0.25, 0.28) | 0.29 (0.27, 0.31) | 31 | 0.034 | 0.042 |
| Chips | 0.072 (0.068, 0.077) | 0.083 (0.073, 0.094) | 0.069 (0.060, 0.077) | 0.066 (0.058, 0.075) | 0.072 (0.064, 0.080) | -13 | 0.001 | 0.001 |
| Savoury snacks | 0.015 (0.014, 0.016) | 0.016 (0.013, 0.018) | 0.014 (0.012, 0.016) | 0.016 (0.013, 0.018) | 0.014 (0.012, 0.016) | -12 | 0.19 | 0.08 |
| Nuts and seeds | 0.014 (0.011, 0.016) | 0.0090 (0.0060, 0.013) | 0.014 (0.0090, 0.019) | 0.012 (0.0080, 0.017) | 0.019 (0.013, 0.025) | 104 | 1.00 | 0.50 |
| Fruit | 0.042 (0.040, 0.045) | 0.030 (0.026, 0.035) | 0.042 (0.037, 0.047) | 0.044 (0.039, 0.048) | 0.053 (0.047, 0.058) | 73 | 0.002 | 0.004 |
| Sugar, preserves, confectionery | 0.0080 (0.0070, 0.0090) | 0.0070 (0.0050, 0.010) | 0.0080 (0.0060, 0.0090) | 0.0090 (0.0060, 0.012) | 0.0080 (0.0070, 0.010) | 15 | 0.96 | 0.24 |
| Non-alcoholic beverages | 0.037 (0.033, 0.041) | 0.037 (0.027, 0.046) | 0.038 (0.031, 0.046) | 0.033 (0.026, 0.041) | 0.039 (0.032, 0.047) | 8 | 1.00 | 0.50 |
| Alcohol | 0.0017 (0.00010, 0.0035) | 0.0035 (0.0012, 0.0082) | 0.00010 (0.0, 0.00020) | 0.0029 (0.0026, 0.0083) | 0.00030 (0.00010, 0.00060) | -93 | 1.00 | 0.45 |
| Miscellaneous | 0.073 (0.064, 0.082) | 0.087 (0.061, 0.11) | 0.069 (0.054, 0.085) | 0.060 (0.050, 0.070) | 0.077 (0.061, 0.092) | -11 | 0.98 | 0.14 |

^1^All values are mean (95% CIs). Values shown for quartiles of total dairy product consumption are min and max grams consumed per day. Data from the National Diet and Nutrition Survey (NDNS) years 1-4 (2008/2009-2011/2012). Q, quartile. ^2^Based on Bonferroni post-hoc test comparing the highest and lowest dairy quartiles. ^3^Differences between dietary thiamin intake from main food groups across dairy quartiles using general linear models adjusted for age, sex and total energy intake.

**Supplemental Table 16.** Riboflavin intake (mg/d) from major food groups across quartiles of total dairy intake^1^

|  |  | Quartiles of dairy product consumption (g/d) | | | |  |  |  |
| --- | --- | --- | --- | --- | --- | --- | --- | --- |
|  |  |  |  |  |  |  |  |  |
| Food group | Total  *n*=1655 | 1  (0-96)  *n*=411 | 2  (97-172)  *n*=410 | 3  (97-172)  *n*=410 | 4  (274-1429)  *n*=420 | Diff Q4/Q1, % | *P*  Q1 vs. Q4^2^ | *P*^3^ |
| Cereals and cereal products | 0.31 (0.30, 0.33) | 0.20 (0.18, 0.22) | 0.28 (0.26, 0.29) | 0.37 (0.34, 0.39) | 0.41 (0.38, 0.44) | 108 | 0.0001 | 0.0001 |
| Milk and milk products | 0.49 (0.47, 0.50) | 0.15 (0.14, 0.16) | 0.32 (0.32, 0.33) | 0.52 (0.51, 0.53) | 0.93 (0.91, 0.96) | 508 | 0.0001 | 0.0001 |
| Eggs and dishes | 0.072 (0.066, 0.078) | 0.078 (0.059, 0.097) | 0.066 (0.057, 0.075) | 0.071 (0.063, 0.080) | 0.073 (0.063, 0.082) | -7 | 0.023 | 0.034 |
| Fat spreads and oils | 0.0053 (0.0044, 0.0061) | 0.0055 (0.0037, 0.0072) | 0.0052 (0.0033, 0.0072) | 0.0048 (0.0032, 0.0065) | 0.0056 (0.0042, 0.0070) | 2 | 1.00 | 0.67 |
| Meat and products | 0.23 (0.22, 0.24) | 0.23 (0.21, 0.25) | 0.24 (0.21, 0.27) | 0.22 (0.20, 0.24) | 0.24 (0.22, 0.26) | 5 | 0.30 | 0.043 |
| Fish and dishes | 0.039 (0.036, 0.041) | 0.039 (0.033, 0.046) | 0.04 (0.034, 0.046) | 0.039 (0.033, 0.044) | 0.037 (0.032, 0.042) | -7 | 0.005 | 0.007 |
| Vegetables and potatoes | 0.088 (0.084, 0.093) | 0.075 (0.069, 0.082) | 0.087 (0.077, 0.098) | 0.090 (0.082, 0.098) | 0.10 (0.091, 0.11) | 32 | 0.36 | 0.28 |
| Chips | 0.014 (0.013, 0.015) | 0.017 (0.014, 0.019) | 0.014 (0.012, 0.016) | 0.013 (0.011, 0.014) | 0.014 (0.012, 0.016) | -14 | 0.058 | 0.022 |
| Savoury snacks | 0.0083 (0.0076, 0.009) | 0.0082 (0.0069, 0.0095) | 0.0085 (0.0070, 0.010) | 0.0088 (0.0071, 0.010) | 0.0078 (0.0064, 0.0091) | -5 | 1.00 | 0.23 |
| Nuts and seeds | 0.0050 (0.0041, 0.0058) | 0.0034 (0.0021, 0.0048) | 0.0049 (0.0033, 0.0065) | 0.0046 (0.0028, 0.0064) | 0.0068 (0.0047, 0.0089) | 98 | 1.00 | 0.62 |
| Fruit | 0.036 (0.034, 0.038) | 0.027 (0.023, 0.031) | 0.034 (0.030, 0.038) | 0.038 (0.034, 0.041) | 0.044 (0.040, 0.049) | 64 | 0.002 | 0.005 |
| Sugar, preserves, confectionery | 0.029 (0.026, 0.032) | 0.023 (0.018, 0.028) | 0.030 (0.024, 0.037) | 0.031 (0.025, 0.037) | 0.030 (0.025, 0.036) | 31 | 1.00 | 0.09 |
| Non-alcoholic beverages | 0.078 (0.073, 0.083) | 0.062 (0.050, 0.074) | 0.072 (0.063, 0.081) | 0.089 (0.080, 0.099) | 0.087 (0.079, 0.095) | 41 | 0.27 | 0.02 |
| Alcohol | 0.078 (0.07, 0.087) | 0.092 (0.074, 0.11) | 0.085 (0.068, 0.10) | 0.065 (0.049, 0.081) | 0.071 (0.054, 0.089) | -23 | 0.0001 | 0.0001 |
| Miscellaneous | 0.071 (0.061, 0.081) | 0.088 (0.059, 0.116) | 0.06 (0.047, 0.073) | 0.060 (0.047, 0.072) | 0.077 (0.059, 0.094) | -13 | 1.00 | 0.12 |

^1^All values are mean (95% CIs). Values shown for quartiles of total dairy product consumption are min and max grams consumed per day. Data from the National Diet and Nutrition Survey (NDNS) years 1-4 (2008/2009-2011/2012). Q, quartile. ^2^Based on Bonferroni post-hoc test comparing the highest and lowest dairy quartiles. ^3^Differences between dietary riboflavin intake from main food groups across dairy quartiles using general linear models adjusted for age, sex and total energy intake.

**Supplemental Table 17.** Vitamin B_12_ intake (μg/d) from major food groups across quartiles of total dairy intake^1^

|  |  | Quartiles of dairy product consumption (g/d) | | | | |  |  |  | |
| --- | --- | --- | --- | --- | --- | --- | --- | --- | --- | --- |
|  |  |  |  |  |  |  |  |  |  |  |
| Food group | Total  *n*=1655 | 1  (0-96)  *n*=411 | 2  (97-172)  *n*=410 | 3  (97-172)  *n*=410 | 4  (274-1429)  *n*=420 | Diff Q4/Q1, % | | *P*  Q1 vs. Q4^2^ | *P*^3^ |  |
| Cereals and cereal products | 0.25 (0.24, 0.27) | 0.18 (0.15, 0.20) | 0.23 (0.20, 0.25) | 0.28 (0.26, 0.31) | 0.31 (0.28, 0.35) | 76 | | 0.0001 | 0.0001 |  |
| Milk and milk products | 1.6 (1.6, 1.7) | 0.50 (0.50, 0.60) | 1.1 (1.1, 1.1) | 1.7 (1.7, 1.8) | 3.0 (2.9, 3.1) | 452 | | 0.0001 | 0.0001 |  |
| Eggs and dishes | 0.34 (0.30, 0.37) | 0.38 (0.26, 0.51) | 0.31 (0.26, 0.35) | 0.33 (0.29, 0.37) | 0.32 (0.28, 0.36) | -16 | | 0.006 | 0.012 |  |
| Fat spreads and oils | 0.090 (0.080, 0.10) | 0.080 (0.060, 0.10) | 0.083 (0.066, 0.099) | 0.089 (0.071, 0.11) | 0.11 (0.086, 0.13) | 35 | | 1.00 | 0.88 |  |
| Meat and products | 1.6 (1.4, 1.7) | 1.5 (1.3, 1.7) | 1.6 (1.3, 2.0) | 1.5 (1.2, 1.8) | 1.6 (1.4, 1.9) | 8 | | 1.00 | 0.43 |  |
| Fish and dishes | 1.0 (0.90, 1.1) | 1.0 (0.90, 1.2) | 1.0 (0.90, 1.1) | 1.0 (0.90, 1.2) | 1.0 (0.80, 1.1) | -5 | | 0.043 | 0.063 |  |
| Vegetables and potatoes | 0.022 (0.018, 0.026) | 0.016 (0.010, 0.022) | 0.025 (0.017, 0.032) | 0.026 (0.015, 0.037) | 0.021 (0.014, 0.028) | 26 | | 1.00 | 0.34 |  |
| Chips | 0.0014 (0.0008, 0.0020) | 0.0007 (0.0003, 0.0012) | 0.0020 (0.0002, 0.0037) | 0.0008 (0.0002, 0.0014) | 0.0020 (0.0004, 0.0036) | 165 | | 1.00 | 0.36 |  |
| Savoury snacks | - | - | - | - | - | - | | - | - |  |
| Nuts and seeds | - | - | - | - | - | - | | - | - |  |
| Fruit | 0.012 (0.0019, 0.027) | 0.021 (0.020, 0.061) | - | 0.025 (0.015, 0.065) | 0.0047 (0.0046, 0.014) | -77 | | 1.00 | 0.57 |  |
| Sugar, preserves, confectionery | 0.034 (0.028, 0.040) | 0.021 (0.015, 0.027) | 0.045 (0.025, 0.065) | 0.037 (0.027, 0.046) | 0.032 (0.024, 0.039) | 47 | | 1.00 | 0.015 |  |
| Non-alcoholic beverages | 0.11 (0.090, 0.14) | 0.14 (0.072, 0.20) | 0.12 (0.070, 0.17) | 0.11 (0.069, 0.15) | 0.089 (0.060, 0.12) | -35 | | 0.47 | 0.33 |  |
| Alcohol | 0.025 (0.017, 0.032) | 0.021 (0.0071, 0.035) | 0.022 (0.0095, 0.034) | 0.020 (0.008, 0.031) | 0.037 (0.015, 0.058) | 75 | | 1.00 | 0.71 |  |
| Miscellaneous | 0.094 (0.072, 0.12) | 0.12 (0.061, 0.19) | 0.069 (0.045, 0.093) | 0.059 (0.04, 0.079) | 0.12 (0.070, 0.18) | 2 | | 1.00 | 0.14 |  |

^1^All values are mean (95% CIs). Values shown for quartiles of total dairy product consumption are min and max grams consumed per day. Data from the National Diet and Nutrition Survey (NDNS) years 1-4 (2008/2009-2011/2012). Q, quartile. ^2^Based on Bonferroni post-hoc test comparing the highest and lowest dairy quartiles. ^3^Differences between dietary vitamin B_12_ intake from main food groups across dairy quartiles using general linear models adjusted for age, sex and total energy intake.

**Supplemental Table 18.** Folate intake (mg/d) from major food groups across quartiles of total dairy intake^1^

|  |  | Quartiles of dairy product consumption (g/d) | | | |  |  |  |
| --- | --- | --- | --- | --- | --- | --- | --- | --- |
|  |  |  |  |  |  |  |  |  |
| Food group | Total  *n*=1655 | 1  (0-96)  *n*=411 | 2  (97-172)  *n*=410 | 3  (97-172)  *n*=410 | 4  (274-1429)  *n*=420 | Diff Q4/Q1, % | *P*  Q1 vs. Q4^2^ | *P*^3^ |
| Cereals and cereal products | 65 (63, 67) | 48 (44, 51) | 59 (56, 62) | 72 (68, 76) | 80 (76, 85) | 69 | 0.0001 | 0.0001 |
| Milk and milk products | 19 (18, 20) | 7.1 (6.5, 7.6) | 14 (13, 14) | 21 (20, 22) | 33 (32, 35) | 374 | 0.0001 | 0.0001 |
| Eggs and dishes | 6.4 (5.9, 7.0) | 6.7 (5.2, 8.3) | 5.9 (5.1, 6.7) | 6.4 (5.7, 7.2) | 6.6 (5.7, 7.5) | -2 | 0.079 | 0.095 |
| Fat spreads and oils | 12 (11, 13) | 11 (8.0, 14) | 11 (9.0, 14) | 12 (9.0, 14) | 14 (11, 17) | 22 | 1.00 | 0.90 |
| Meat and products | 14 (14, 15) | 15 (13, 17) | 14 (12, 15) | 14 (12, 15) | 16 (13, 18) | 2 | 1.00 | 0.34 |
| Fish and dishes | 3.8 (3.5, 4.1) | 4.2 (3.5, 4.9) | 3.5 (2.9, 4.1) | 3.8 (3.2, 4.4) | 3.5 (3.0, 4.0) | -17 | 0.004 | 0.009 |
| Vegetables and potatoes | 62 (60, 64) | 54 (50, 58) | 61 (57, 65) | 64 (60, 68) | 69 (65, 73) | 28 | 0.18 | 0.18 |
| Chips | 11 (10, 12) | 13 (12, 15) | 11 (9.0, 12) | 10 (9.0, 11) | 11 (10, 12) | -18 | 0.0001 | 0.0001 |
| Savoury snacks | 2.7 (2.5, 2.9) | 2.7 (2.3, 3.1) | 2.6 (2.2, 3.0) | 2.8 (2.4, 3.2) | 2.6 (2.2, 3.0) | -3 | 0.90 | 0.36 |
| Nuts and seeds | 1.8 (1.5, 2.1) | 1.3 (0.9, 1.7) | 1.8 (1.3, 2.4) | 1.8 (1.2, 2.3) | 2.3 (1.7, 2.9) | 79 | 1.00 | 0.70 |
| Fruit | 10 (9.3, 11) | 7 (5.9, 8.1) | 10 (8.6, 11) | 11 (9.5, 12) | 12 (11, 13) | 72 | 0.009 | 0.010 |
| Sugar, preserves, confectionery | 1.2 (1.0, 1.3) | 1 (0.8, 1.3) | 1.1 (0.9, 1.3) | 1.3 (1.0, 1.6) | 1.2 (1.0, 1.4) | 18 | 0.84 | 0.24 |
| Non-alcoholic beverages | 14 (13, 15) | 12 (10, 15) | 14 (12, 16) | 15 (12, 17) | 16 (14, 18) | 28 | 1.00 | 0.72 |
| Alcohol | 19 (17, 21) | 23 (18, 27) | 21 (16, 26) | 15 (11, 20) | 17 (12, 22) | -25 | 0.0001 | 0.0001 |
| Miscellaneous | 14 (12, 15) | 15 (11, 18) | 13 (10, 16) | 13 (9.4, 16) | 14 (11, 16) | -6 | 1.00 | 0.56 |

^1^All values are mean (95% CIs). Values shown for quartiles of total dairy product consumption are min and max grams consumed per day. Data from the National Diet and Nutrition Survey (NDNS) years 1-4 (2008/2009-2011/2012). Q, quartile. ^2^Based on Bonferroni post-hoc test comparing the highest and lowest dairy quartiles. ^3^Differences between dietary folate intake from main food groups across dairy quartiles using general linear models adjusted for age, sex and total energy intake.
